# Colorless Polyimides with Low Linear Coefficients of Thermal Expansion and Their Controlled Soft Adhesion/Easy Removability on Glass Substrates: Role of Modified One-Pot Polymerization Method

**DOI:** 10.3390/polym17131887

**Published:** 2025-07-07

**Authors:** Masatoshi Hasegawa, Takehiro Shinoda, Kanata Nakadai, Junichi Ishii, Tetsuo Okuyama, Kaya Tokuda, Hiroyuki Wakui, Naoki Watanabe, Kota Kitamura

**Affiliations:** 1 Department of Chemistry, Faculty of Science, Toho University, 2-2-1 Miyama, Funabashi, Chiba 274-8510, Japan; 2 Toyobo Co., Ltd., Corporate Research Center, 1-1, Katata 2-chome, Otsu-shi, Shiga 520-0292, Japan

**Keywords:** colorless polyimides, optical transparency, heat resistance, linear coefficients of thermal expansion (CTE), water uptake, toughness, controlled solid–solid adhesion, easy delamination

## Abstract

This study presents colorless polyimides (PIs) suitable for use as plastic substrates in flexible displays, designed to be compatible with controlled soft adhesion and easy delamination (temporary adhesion) processes. For this purpose, we focused on a PI system derived from norbornane-2-spiro-α-cyclopentanone-α′-spiro-2″-norbornane-5,5″,6,6″-tetracarboxylic dianhydride (CpODA) and 2,2′-bis(trifluoromethyl)benzidine (TFMB). This system was selected with the aim of exhibiting excellent optical transparency and low linear coefficient of thermal expansion (CTE) properties. However, fabricating this PI film via the conventional two-step process was challenging because of crack formation. In contrast, modified one-pot polymerization at 200 °C using a combined catalyst resulted in a homogeneous solution of PI with an exceptionally high molecular weight, yielding a flexible cast film. The solubility of PI plays a crucial role in its success. This study delves into the mechanism behind the significant catalytic effect on enhancing molecular weight. The CpODA/TFMB PI cast film simultaneously achieved very high optical transparency, an extremely high glass transition temperature (*T*_g_ = 411 °C), a significantly low linear coefficient of thermal expansion (CTE = 16.7 ppm/K), and sufficient film toughness, despite the trade-off between low CTE and high film toughness. The CpODA/TFMB system was modified by copolymerization with minor contents of another cycloaliphatic tetracarboxylic dianhydride, 5,5′-(1,4-phenylene)-*exo*-bis(hexahydro-4,7-methanoisobenzofuran-*cis-exo*-1,3-dione) (BzDAxx). This approach was effective in improving the film toughness without sacrificing the low CTE and other target properties. The peel strengths (*σ*_peel_) of laminates comprising surface-modified glass substrates and various colorless PI films were measured to evaluate the compatibility with the temporary adhesion process. Most colorless PI films studied were found to be incompatible. Additionally, no correlation between *σ*_peel_ and PI structure was observed, making it challenging to identify the structural factors influencing *σ*_peel_ control. Surprisingly, a strong correlation was observed between *σ*_peel_ and CTE of the PI films, suggesting that the observed solid–solid lamination is closely linked to the unexpectedly high surface mobility of the PI films. The laminate using CpODA(90);BzDAxx(10)/TFMB copolymer exhibited suitable adhesion strength for the temporary adhesion process, while meeting other target properties. The modified one-pot polymerization method significantly contributed to the development of colorless PIs suitable for plastic substrates.

## 1. Introduction

In flat panel displays, such as liquid-crystal displays and organic light-emitting diode displays, non-alkali-glass substrates have been preferred due to their excellent properties, including optical transparency, heat resistance, and dimensional stability against multiple heating–cooling cycles during device fabrication [1]. However, glass substrates are not ideal for reducing weight and making display devices more flexible. Therefore, colorless plastic substrates play a crucial role in developing lightweight and flexible displays [2,3]. However, current colorless resins face challenges in direct application to plastic substrates due to their limited short-term heat resistance [glass transition temperature (*T*_g_)] and poor thermal dimensional stability. For instance, even poly(ether sulfone) with a relatively high *T*_g_ (225 °C) [4] is not suitable for the severe thermal conditions encountered during flexible display manufacturing processes. The practical polymeric materials with exceptionally high heat resistance required for this purpose are primarily limited to wholly aromatic polyimides (PIs) [5,6,7,8,9,10,11,12,13] or polybenzoxazoles (PBOs) [14,15,16]. PIs are preferred over PBOs due to the simplicity of the film manufacturing process and the availability of monomers from commercial sources, which are essential for structural modifications. Therefore, with advancements in performance enhancement, functionalization, miniaturization, weight reduction, and flexibilization of electronic devices, the chemistry, physics, properties, characterization techniques, and applications of PIs have been extensively investigated in both industry and academia [17,18,19,20,21,22,23,24,25].

However, optical and optoelectronic applications (e.g., the use as transparency-emphasizing plastic substrates) of conventional aromatic PI films are significantly limited by their intense coloration due to charge-transfer (CT) interactions [26], as observed in the commercially available PI films KAPTON^®^ H [27] and UPILEX^®^-S [28]. Colorless PIs offer a potential solution to this issue. Among wholly aromatic PIs, colorless options are primarily restricted to a fluorinated PI system derived from 4,4′-(hexafluoroisopropylidene)diphthalic anhydride (6FDA) and 2,2′-bis(trifluoromethyl)benzidine (TFMB). However, this PI film lacks excellent thermal dimensional stability [29,30], a crucial property for plastic substrates in flexible devices. Inadequate thermal dimensional stability poses risks during the multiple heating–cooling cycles in device fabrication, such as misalignment, adhesion failure of various microcomponents, laminate warpage, and breakdown of transparent electrodes. Therefore, plastic substrates must exhibit significantly higher *T*_g_s than the maximum processing temperature and a low linear coefficient of thermal expansion (CTE < ~20 ppm/K) in the *XY*-direction within the glassy temperature region (*T* < *T*_g_).

**Figure 1:**
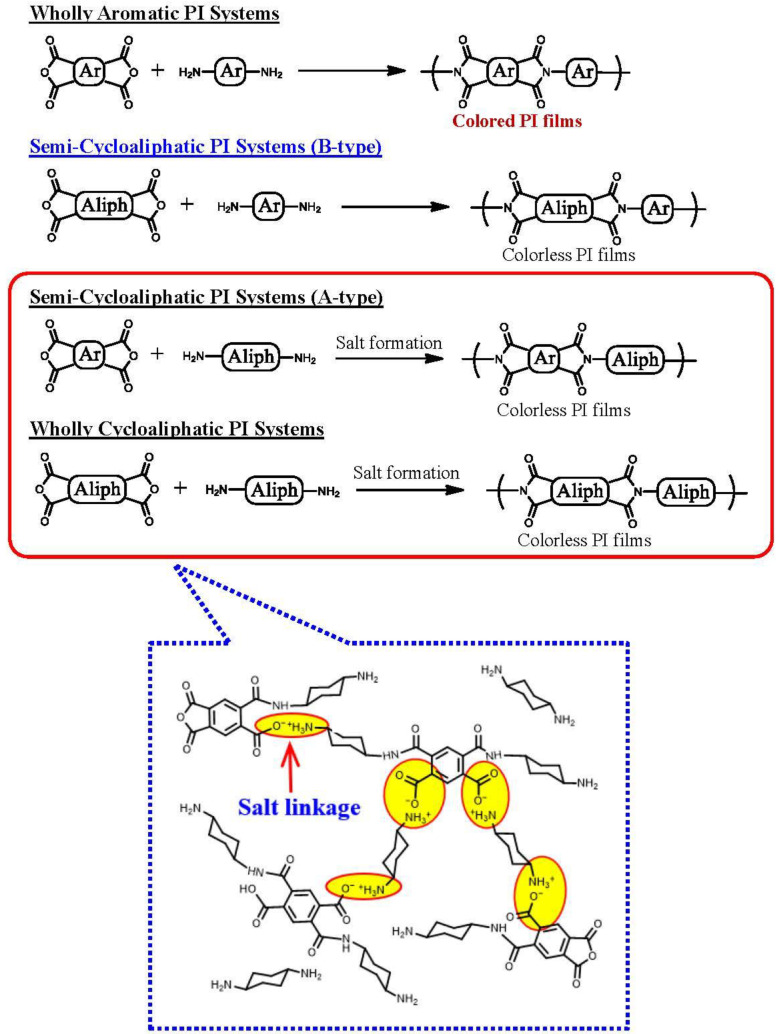
Basic structures of three types of PIs (wholly aromatic, semi-cycloaliphatic (A- and B-type), and wholly cycloaliphatic PIs) and a schematic illustration of predicted crosslink structure based on salt formation in the systems using cycloaliphatic diamines (a typical system consisting of pyromellitic dianhydride (PMDA) with *trans*-1,4-cyclohexanediamine (*t*-CHDA) is depicted here).

In general, the coloration of PI films can be removed by selecting aliphatic monomers (typically, cycloaliphatic monomers to prevent a significant decrease in the heat resistance of the PIs). This can be achieved by using either tetracarboxylic dianhydrides (TCDAs) or diamines to hinder CT interactions [26], as shown in Figure 1. Numerous semi- and wholly cycloaliphatic PIs have been investigated [31,32,33,34,35,36,37,38,39,40,41,42,43,44,45,46,47,48,49,50,51,52,53,54,55,56,57,58,59,60,61,62,63,64,65]. However, the use of cycloaliphatic diamines led to a disruption in the smooth progression of the polyaddition due to the formation of insoluble salt at the initial reaction stage [48] (Figure 1). In contrast, the semi-cycloaliphatic PI systems derived from cycloaliphatic TCDAs and aromatic diamines (we will call this “B-type” in this paper) are considered valuable in practice as they avoid salt formation during the polyaddition of cycloaliphatic TCDAs and aromatic diamines [64].

**Figure 2:**
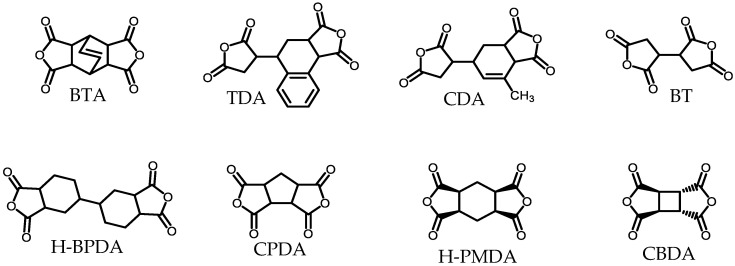
Molecular structures and abbreviations of common cycloaliphatic tetracarboxylic dianhydrides.

However, commercially available cycloaliphatic TCDAs are not so abundant, as shown in Figure 2, which exhibits their typical products. This limitation poses a challenge to widespread structural modifications. Industrially produced cycloaliphatic TCDAs with high or acceptable polyaddition reactivity with aromatic diamines are primarily limited to hydrogenated pyromellitic dianhydride (1-*exo*,2-*exo*,4-*exo*,5-*exo*-cyclohexanetetracarboxylic dianhydride, H-PMDA) and 1,2,3,4-cyclobutanetetracarboxylic dianhydride (CBDA) [36]. These have merits and demerits; H-PMDA is advantageous for producing highly soluble PIs due to its non-planar/non-linear steric structure. However, the resulting PI films often lack low CTE characteristics [56]. In contrast, a relatively linear/rigid crank-shaft-like structure of CBDA helps reduce CTE but hinders the formation of highly tough PI films [65]. Cycloaliphatic TCDAs often do not exhibit high polyaddition reactivity with aromatic diamines, leading to insufficient molecular weights (*M*_w_s) of the PI precursors [poly(amic acid)s, PAAs] for subsequent film preparation. For instance, a system derived from H-PMDA and a fluorinated diamine yielded a cracked PI film [56] in the conventional two-step process, similar to a related system using 5,5′-bis(2,3-norbornandicarboxylic anhydride) (BNBDA) [64]. Consequently, the difficulty in obtaining crack-free ductile PI films due to the *M*_w_ issues has hindered the exploration of various features of PIs from different cycloaliphatic TCDAs. Therefore, the importance of a well-refined polymerization method and film preparation process as an alternative to the conventional two-step process has significantly increased to dramatically enhance somehow the *M*_w_s of PIs. This study delves into identifying cycloaliphatic TCDAs suitable for this purpose and thoroughly investigates the optimal polymerization/filming conditions to develop new colorless PIs with prominent features. As a result, we focused on norbornane-2-spiro-α-cyclopentanone-α′-spiro-2″-norbornane-5,5″,6,6″-tetracarboxylic dianhydride (CpODA) because the combination with 2,2′-bis(trifluoromethyl)benzidine (TFMB) has the potential to meet the target properties if a highly flexible film of this PI is successfully obtained.

**Figure 3:**
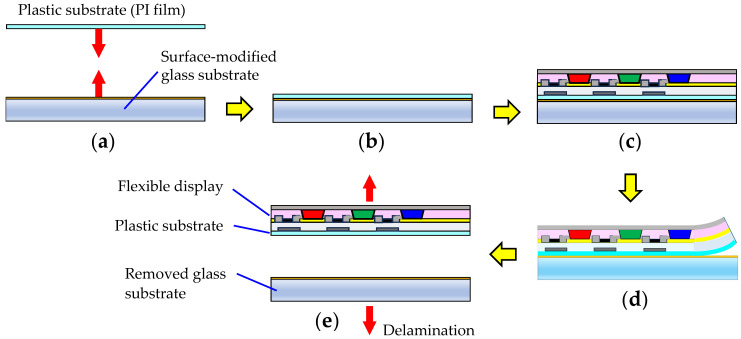
Schematic diagram for the controlled soft adhesion/post-delamination process in flexible display fabrication: (**a**) a set of PI film and surface-modified glass substrate, (**b**) roll-laminated PI film and glass substrate with controlled soft adhesion, (**c**) electronic component mounting, (**d**) easy post-delamination, and (**e**) removal of the glass substrate and completed flexible devices.

This study also examined the compatibility of the developed PI films with a controlled soft adhesion/easy removal process on glass-carrier substrates. This technique was devised for the temporary lamination of a super-heat-resistant film (XENOMAX_®_ [66]) and surface-modified glass substrates. The laminates maintain sufficient adhesion during device assembly and allow for easy peeling without any triggers [67,68] (see Figure 3), unlike the current laser lift-off (LLO) method based on interfacial ablation [69], which is effective for peeling tightly adhered laminates. The key advantages of this easy peeling method include not requiring expensive facilities such as the LLO method and avoiding ash generation and damage to the PI surface from high-power laser scanning. However, PI films are not always compatible with this easy peeling method due to significantly deviated adhesion strengths from its optimum value. Furthermore, the relationship between the PI structure and the adhesion strength of solid–solid laminates is not well understood. This study also discusses a hidden parameter for controlling the adhesion strength of the laminates.

## 2. Experimental Section

### 2.1. Materials

#### 2.1.1. Monomers

**Figure 4:**
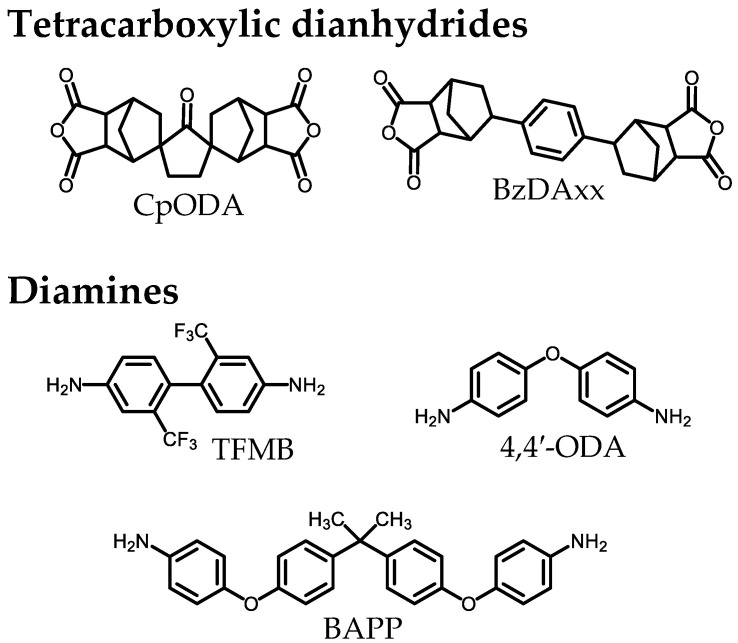
Molecular structures and abbreviations of the monomers used in this study.

The molecular structures of the monomers used in this study are shown in Figure 4. Their commercial sources, abbreviations, pre-drying conditions, and melting points are listed in Table 1.

**Scheme 1:**
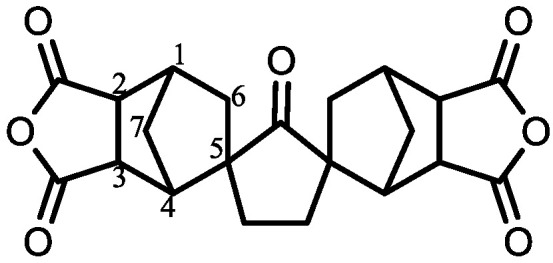
Molecular structure of CpODA with numbering.

The analytical data of CpODA (Scheme 1) are as follows. FT-IR (KBr plate method, cm^−1^): 2960/2948/2865/2852 (C_aliph_–H), 1861/1783 (dicarboxylic acid anhydride, C=O), 1721 (central C=O). ^1^H-NMR (400 MHz, CDCl_3_, *δ*, ppm): 3.15 [d, 2H (relative integrated intensity: 1.98H), *J* = 7.7 Hz, 3,3′-protons of the norbornane (NB) units], 2.94 [d, 2H (2.03H), *J* = 7.7 Hz, 2,2′-protons of NB], 2.89 [d, 2H (1.97H), *J* = 4.2 Hz, 4,4′-protons of NB], 2.66 [s, 2H, 1,1′-protons of NB], 2.35 [d, 2H (2.05H), *J* = 11.9 Hz, 6(H_a_),6′(H_a_)-protons of NB], 1.98–1.78 [m, 6H (5.96H), the cyclopentane unit (4H) + 7(H_a_),7′(H_a_)-protons of NB (2H)], 1.33 [d, 2H (1.95H), *J* = 11.8 Hz, 6(H_b_),6′(H_b_)-protons of NB], 1.26 [dd, 2H (2.09H), *J* = 12.7, 2.8 Hz, 7(H_b_),7′(H_b_)-protons of NB]. Elemental analysis, Anal. Calcd. (%) for C_21_H_20_O_7_ (384.39): C, 65.62; H, 5.24. Found: C, 65.30; H, 5.20.

**Scheme 2:**
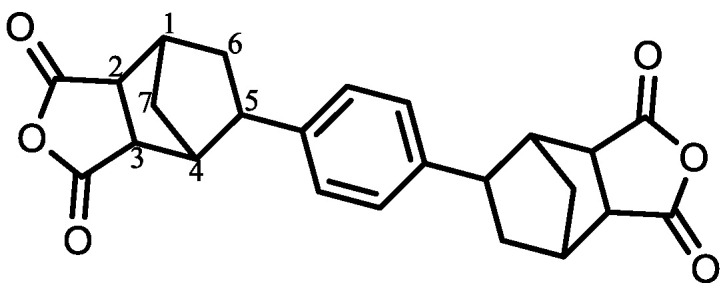
Molecular structure of BzDAxx with numbering.

The analytical data of BzDAxx (Scheme 2) are as follows. Melting point (DSC): 209 °C (broad endothermic peak). FT-IR (KBr plate method, cm^−1^): 3035 (C_arom_–H stretching), 2995/2975/2884 (C_aliph_–H), 1838/1774 (dicarboxylic acid anhydride, C=O). 1517/1476 (1,4-phenylene unit). ^1^H-NMR (400 MHz, dimethyl sulfoxide (DMSO)-*d*_6_, *δ*, ppm): 7.20 [s, 4H (4.00H), the 1,4-phenylene unit], 3.28 [d, 2H (2.02H), *J* = 7.3 Hz, 3,3′-protons of the norbornane (NB) units], 3.14 [d, 2H (2.01H), *J* = 7.5 Hz, 2,2′-protons of NB], 2.97–2.93 [m, 2H (2.02H), 5,5′-protons of NB], 2.72 [d, 2H (2.01H), *J* = 3.8 Hz, 4,4′-protons of NB], 2.60 [s, 2H (2.02H), 1,1′-protons of NB], 1.94–1.88 [m, 2H (2.00H), 6(H_a_),6′(H_a_)-protons of NB], 1.77–1.71 [m, 2H (2.06H), 6(H_b_),6′(H_b_)-protons of NB], 1.54 [d, 2H (2.05H), *J* = 11.6 Hz, 7(H_a_),7′(H_a_)-protons of NB], 1.10 [d, 2H (1.99H), *J* = 11.5 Hz, 7(H_b_),7′(H_b_)-protons of NB]. Elemental analysis, Anal. Calcd. (%) for C_24_H_22_O_6_ (406.44): C, 70.92; H, 5.46. Found: C, 70.66; H, 5.30.

#### 2.1.2. Model Compounds

CpODA- and BzDAxx-based diimide model compounds (Schemes S1 and S2) were synthesized and characterized, as described in Documents S1 and S2.

The comparisons of the ^1^H-NMR spectra for these model compounds and those of their staring materials (CpODA and BzDAxx) suggest that the steric structures of the latter were maintained during the addition reaction with aniline and subsequent thermal imidization of the resultant amic acid in solution.

#### 2.1.3. Polymerization and Film Preparation

**Figure 5:**
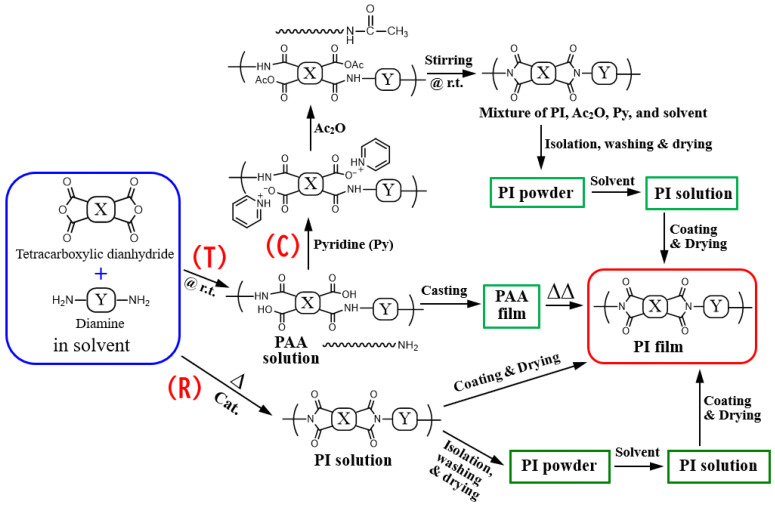
Schemes of polymerization, imidization, and film preparation via different pathways: (**T**) conventional two-step process, (**C**) chemical imidization process, and (**R**) modified one-pot process.

In this study, the PI films were prepared via the following three processes, as shown in Figure 5; Route T: a conventional two-step process consisting of low-temperature equimolar polyaddition, coating/drying of the resultant solutions of PI precursors [poly(amic acid)s, PAAs], and thermal imidization of the PAA cast films at elevated temperatures. Route C: chemical imidization by adding a cyclodehydration reagent [acetic anhydride (Ac_2_O)/pyridine (7/3, *v*/*v*)] with a fixed molar ratio of [Ac_2_O]/[COOH]_PAA_ = 5 to the PAA solutions (diluted as appropriate), isolation of PIs, re-dissolution of the isolated fibrous PI powder in a fresh solvent, and coating/drying of the resultant homogeneous PI solutions [54]. Route R: modified one-pot PI polymerization by refluxing the monomer mixtures in solution in the presence of catalysts and coating/drying of the PI solutions, which are either the as-polymerized PI solutions or the catalyst-removed PI solutions prepared via isolation and re-dissolution.

The modified one-pot polymerization (Route R) was conducted as described in our previous papers [56,58,64]; the reaction apparatus comprises a specially designed small-capacity separable vessel, a dry nitrogen gas inlet and outlet connected to a silicone oil-sealed bubbler, condenser, Dean–Stark trap, sealed mechanical stirrer with a non-contact magnetic coupling mechanism (Nakamura Scientific Instruments Industry, Tokyo, Japan, UZ-SM1), and temperature-controllable oil bath. A typical procedure of the modified one-pot polymerization process is as follows. Diamine (5 mmol) and benzoic acid (BA, 10 mmol, 1 equivalent (Eq.)) as a cocatalyst was completely dissolved in a selected dehydrated solvent [*γ*-butyrolactone (GBL), *N*,*N*-dimethylacetamide (DMAc), or *N*-methyl-2-pyrrolidone (NMP)] in the reaction vessel at room temperature (or by mildly warming as appropriate) with continuous mechanical stirring. To this solution, 1-ethylpiperidine (1-EP, 10 mmol, 1 Eq.) as a catalyst and subsequently tetracarboxylic dianhydride (TCDA) powder (5 mmol) were added in one portion. The reaction mixture in the sealed reaction vessel was then rapidly heated by immersing the vessel into the oil bath maintained at an established elevated temperature and refluxed at the boiling point of the solutions for 4 h in an N_2_ atmosphere with continuous mechanical stirring. The reaction was started at a total monomer content of 40 or 50 wt%, and the reaction mixture was gradually diluted as appropriate with a minimum amount of the same solvent for ensuring effective mixing.

The resultant homogeneous PI solutions containing the catalyst residue can be directly used for the subsequent solution casting. However, in this study, PIs were isolated by gradually dripping the adequately diluted PI solution into a large quantity of methanol as a poor solvent, followed by filtering out of the precipitate, thorough washing with methanol, and drying the white fibrous precipitate at 120 °C for 12 h under vacuum. This procedure completely removed the catalyst residue and the solvents.

**Figure 6:**
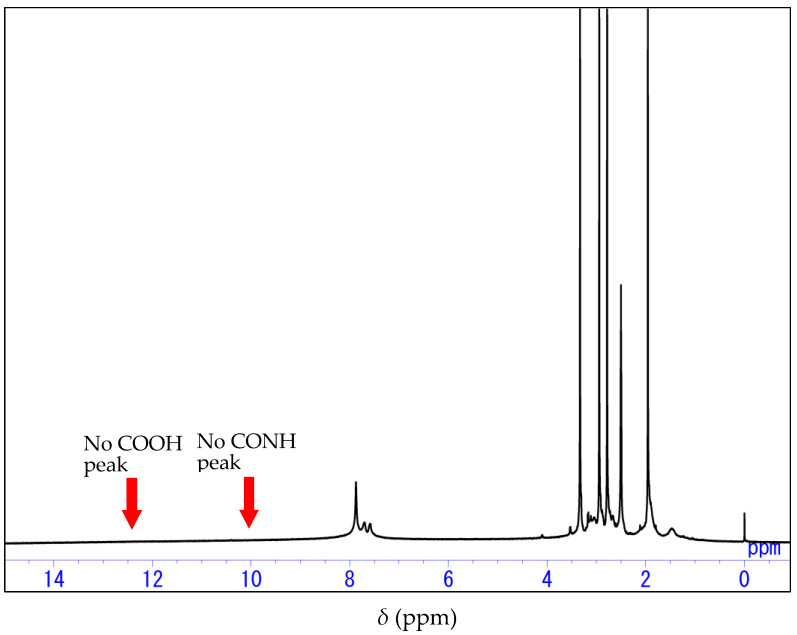
^1^H-NMR spectrum (400 MHz, DMSO-*d*_6_) of CpODA/TFMB obtained via modified one-pot polymerization.

A typical transmission-mode FT-IR spectrum of PI thin film (CpODA/TFMB) obtained via the modified one-pot method is shown in Figure S1. The spectrum includes the specific bands (cm^−1^): 2959/2891/2870 (C_aliph_–H), 1777 (imide, C=O), 1715 (imide + cyclopentanone, C=O), 1491 (1,4-phenylene), 1371 (imide, N–C_arom_), 1313/1173 (CF_3_, C–F). In addition, the PAA-specific bands at ~2600 cm^−1^ (hydrogen-bonded COOH, O–H stretching) and 1680/1530 cm^−1^ (amide, C=O stretching) completely disappear. A typical ^1^H-NMR spectrum (400 MHz, DMSO-*d*_6_) of the PI prepared via the modified one-pot method is shown in Figure 6. No NHCO and COOH proton signals due to the amide acid units were observed. These spectra confirmed the completion of imidization by the modified one-pot polymerization.

The isolated fibrous PI powder after the modified one-pot polymerization process was re-dissolved in a fresh solvent at a high solid content (typically, 15 wt%), and the resultant homogeneous PI solutions were coated on a glass substrate and soft-dried at 65 °C for 2 h in an air convention oven. The PI films (typically, 20 μm thick) were then heated typically at 150 °C/0.5 h + 200 °C/0.5 h + 250 °C/1 h on the substrates under vacuum and additionally annealed at 300 °C/1 h under vacuum in a tube furnace without the substrates to remove residual strain in the films. These thermal conditions were adjusted to obtain better-quality films when the films were very brittle.

In this study, the PAA and PI systems were represented using the abbreviations of the monomer symbols [tetracarboxylic dianhydrides (A) and diamines (B)] as A/B for homopolymers and A1;A2/B1;B2 for copolymers.

### 2.2. Measurements

#### 2.2.1. Structural Characterization

CpODA, BzDAxx, and the related low-*M*_w_ diimide model compounds were characterized by FT-IR (KBr plate method, JASCO, Tokyo, Japan, FT/IR 4100 infrared spectrometer) and ^1^H-NMR spectra in DMSO-*d*_6_ or CDCl_3_ (JEOL, Tokyo, Japan, ECP400). Complete imidization for the PIs obtained via the chemical imidization (C) and the modified one-pot polymerization process (R) was confirmed by the ^1^H-NMR spectra in DMSO-*d*_6_ and transmission-mode FT-IR spectra of their thin films (~5 μm thick) with an intentionally roughened surface to remove interference fringes. The melting points of the monomers were determined by differential scanning calorimetry (DSC, Rigaku, Tokyo, Japan, DSC 8231) as the endothermic peak temperatures in the DSC thermograms measured at a heating rate of 5 °C min^−1^ in a nitrogen atmosphere.

#### 2.2.2. Inherent Viscosities and Molecular Weights

A polyelectrolyte effect of PAAs disturbs the determination of their inherent viscosities (*η*_inh_) by extrapolation to zero concentration. Thus, in this study, instead of *η*_inh_, the reduced viscosities (*η*_red_) of PAAs and/or PIs were measured at a low polymer concentration of 0.5 wt% at 30 °C on an Ostwald viscometer. The measurements were conducted promptly after dilution of the as-polymerized PAA solutions to 0.5 wt% because the dilution of PAA solutions to a low concentration (<1 wt%) often accelerates their molecular weight decrease.

The number-(*M*_n_) and weight-(*M*_w_) average molecular weights for soluble PIs in tetrahydrofuran (THF) at 0.05 wt%, of which the dilute solution was pre-filtered with a PTFE-membrane filter with a pore size of 0.1 μm, were estimated by gel permeation chromatography (GPC) using THF as an eluent at room temperature on a HPLC system (Jasco, Tokyo, Japan, LC-2000 Plus) equipped with a GPC column (Resonac, Tokyo, Japan, Shodex, KF-806L) and ultraviolet–visible detector set at 300 nm (Jasco, Tokyo, Japan, UV-2075) at a flow rate of 1 mL min^−1^. The calibration was performed using standard polystyrenes (Shodex, SM-105).

#### 2.2.3. Linear Coefficients of Thermal Expansion (CTE)

The thermal dimensional stability of the PI films in the *XY*-direction was evaluated based on their CTE values. Thermomechanical analysis (TMA) was conducted to determine the *XY*-direction CTEs of the PI films (specimen length: 20 mm, width: 5 mm, typical thickness: 20 μm, and chuck-to-chuck length: 15 mm) using a thermomechanical analyzer (Rigaku, Tokyo, Japan, TMA 8311). The CTEs were measured at a heating rate of 5 °C min^−1^ with a fixed load (0.5 g per unit film thickness in μm, i.e., 10 g-load for 20 μm-thick films) in a dry nitrogen atmosphere and calculated as an average between 100 and 200 °C in the glassy temperature region (*T* < *T*_g_). TMA data were obtained from the second heating run ranging from 30–450 °C after the tentative first run up to 150 °C to eliminate adsorbed water on the films, followed by cooling to room temperature under dry N_2_ flow in the sealed TMA chamber.

The following factors of the film specimens should be considered to appropriately measure the CTEs.

(1) Residual strain;
(2) Adsorbed water;
(3) Incomplete imidization and residual solvents;
(4) Crystallization and orientational relaxation (at *T* > *T*_g_).

Factor (3) based on the use of “undercooked” PI samples causes film shrinkage during the TMA heating run. Thus, this sample deficiency should be thoroughly examined beforehand through spectroscopic and thermogravimetric analyses. Film shrinkage resulting from factor (4) can also occur during the heating run, leading to significantly deformed TMA curves rather than an ideal straight line. These irreversible phenomena typically manifest above *T*_g_s.

The adsorbed water [factor (2)] can also contribute to film shrinkage during desorption around 100 °C in the first heating run. Therefore, the CTEs should be calculated using the TMA second heating run data, which was conducted after complete water desorption in the preliminary first run up to 150 °C under a dry N_2_ atmosphere.

Factor (1) is identified as the most essential. As-prepared PI films upon thermal imidization (or solution casting) on substrates often include residual strain, leading to film shrinkage by strain relaxation in the TMA heating process. Annealing without substrates at a temperature slightly above the final imidization temperature (*T*_i_) effectively eliminates this residual strain. For thermoplastic PI films, residual strain removal can be achieved by annealing without substrates at a temperature approximately 20 °C lower than the *T*_g_ to prevent film deformation unfavorable for subsequent testing. The complete removal of residual strain is validated by the reversibility observed in the thermal expansion (heating) and contraction (cooling) processes within the range of 100–200 °C (<*T*_g_), where the heating and cooling TMA curves almost overlay with nearly identical slopes. The accurately measured CTEs often exhibit a strong correlation with thickness-direction birefringence (Δ*n*_th_), particularly when comparing the same type of PIs, such as semi-cycloaliphatic PIs (B-type).

#### 2.2.4. Heat Resistance

Dynamic mechanical thermal analysis (DMA) was conducted to determine the *T*_g_s of the PI films as their short-term (physical) heat resistance on a dynamic viscoelastic analyzer (TA Instruments Japan, Tokyo, Japan, DMA-Q800). The storage modulus (*E*′) and loss modulus (*E*″) were measured in the temperature range of 30–450 °C at a heating rate of 5 °C/min in an N_2_ atmosphere under a sinusoidal strain frequency of 0.1 Hz (amplitude: 0.1%). The *T*_g_s were determined from the peak temperatures in the *E*″ curve, unless otherwise noted. A typical DMA curve is shown in Figure S2.

The *T*_g_s of the PI films were also measured by TMA from an inflection point (abrupt softening temperature) in the TMA curve, determined from an intersection of two tangential lines. A typical TMA curve is shown in Figure S3. The TMA-based *T*_g_s were essentially equivalent to the DMA-based values, although the former tended to be 5–20 °C higher than the latter.

Thermogravimetric analysis (TGA) was conducted to evaluate the thermal and thermo-oxidative stability of the PI films from the 5% weight loss temperatures (*T*_d_^5^) measured at a heating rate of 10 °C min^−1^ in a dry nitrogen and/or air atmosphere. A small weight loss due to the desorption of adsorbed water at 100 °C during the TGA heating runs was compensated by an off-set at 150 °C for the data analysis.

#### 2.2.5. Optical Transparency

Optical transparency of the PI films (typically 20 μm thick) was evaluated from the light transmittance at 400 nm (*T*_400_), cut-off wavelength (*λ*_cut_), yellowness indices (YI), total light transmittance (*T*_tot_), and haze. The optical transmission spectra were measured in the wavelength (*λ*) range from 200–800 nm to determine the *T*_400_, *λ*_cut_, and YI on an ultraviolet–visible spectrophotometer (JASCO, Tokyo, Japan, V-530). The YI values were calculated under a standard illuminant of D65 and a standard observer function of 2° (ASTM E 313) using color calculation software (JASCO, Tokyo, Japan) on the basis of the following relationship:

YI = 100 × (1.2985*x* − 1.1335*z*)/*y* (1)

where *x*, *y*, and *z* are the CIE tristimulus values. YI becomes zero for an ideal white/transparent sample. The *T*_tot_ (JIS K 7361-1) and diffuse transmittance (*T*_diff_, JIS K 7136) of PI films were measured on a double-beam haze meter equipped with an integrating sphere (Nippon Denshoku Industries, Tokyo, Japan, NDH 4000), as described in our previous paper [64].The haze of the PI films was calculated from the following relationship:

Haze = (*T*_diff_/*T*_tot_) × 100 (2)

#### 2.2.6. Birefringence and Optically Estimated Dielectric Constant

The thickness-direction birefringence (Δ*n*_th_) of the PI films was measured to estimate a relative extent of chain alignment to the film plane at 589.3 nm (Na lamp, *D*-line) on an Abbe refractometer (ATAGO, Tokyo, Japan, 4T, *n*_D_ range: 1.47–1.87) with a polarizer using a contact liquid (sulfur-saturated methylene iodide, *n*_D_ = 1.78–1.80) and a test piece (*n*_D_ = 1.92). The Δ*n*_th_ values were calculated from the following relationship:

Δ*n*_th_ = *n*_in_ − *n*_out_ (3)

where *n*_in_ and *n*_out_ denote the in-plane and out-of-plane refractive indices of the PI films. This parameter is closely related to the extent of polymer chain alignment along the *XY*-(film plane) direction (called “in-plane chain orientation”) [70,71].

The optically estimated dielectric constants (*ε*_opt_) for the PI films were calculated from the following empirical relationship [37]:

*ε*_opt_ = 1.1 × *n*_av_^2^ (4)

here, *n*_av_ denotes the average refractive indices [*n*_av_ = (2*n*_in_ + *n*_out_)/3] of the PI films.

#### 2.2.7. Mechanical Properties

The mechanical properties [tensile modulus (*E*), tensile strength (*σ*_b_), and elongation at break (*ε*_b_)] of the PI films (specimen length: 30 mm, width: 3 mm, typical thickness: 20 μm, and number of valid specimens > 10) were measured at a cross head speed of 8 mm min^−1^ at room temperature on a mechanical testing machine (A & D, Tokyo, Japan, Tensilon UTM-II). The raw data were analyzed using a data processing program (Softbrain, Tokyo, Japan, UtpsAcS Ver. 4.09).

#### 2.2.8. Water Uptake

The water uptake (*W*_A_) of the PI films was determined from the following relationship:

*W*_A_ = [(*W* − *W*_0_)/*W*_0_] × 100 (5)

where *W*_0_ is the weight of a vacuum-dried film sample at 50 °C for 24 h and *W* is the weight of the film immersed in water at 23 °C for 24 h and carefully blotted dry with tissue paper. KAPTON^®^ H film (Toray-DuPont, Tokyo, Japan, thickness: 50 μm) was used as a reference sample (*W*_A_ = 2.7% [27]) in each weighing. The static electricity of the specimens was discharged using a static elimination gun (Milty, Bishop’s Stortford, UK, Zerostat 3) before each weighing.

#### 2.2.9. Interfacial Adhesion Strength of PI Film/Glass Substrate Laminates

**Figure 7:**
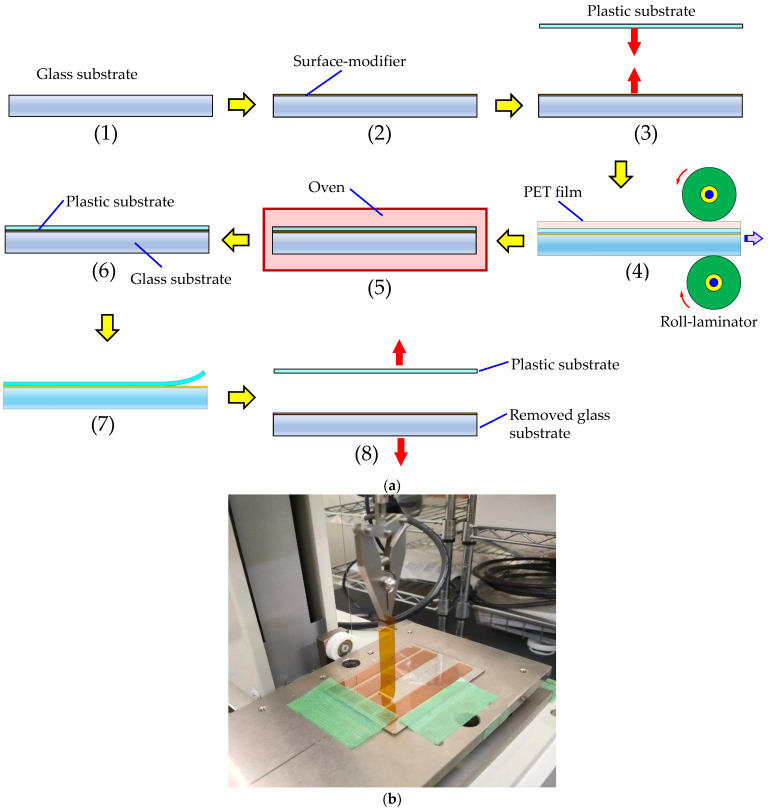
Flexible device-imitated testing for the temporary soft adhesion/post-delamination process: (**a**) specimen preparation steps: (1) pre-cleaned non-alkali glass substrate, (2) surface-modified glass substrate with silane coupling agent, (3) a set of PI film and the surface-modified glass substrate, (4) roll-lamination at room temperature, (5) mild heating of the laminate, (6) the specimens completed, (7) peel testing, and (8) removal of the glass substrate; and (**b**) appearance of the 90° peel testing.

PI film/glass substrate laminates were prepared according to the procedure outlined in Figure 7a [67,68]. Non-alkali glass substrates (dimensions: 8 cm × 8 cm × 0.5 mm-thickness, Nippon Electric Glass, Otsu, Japan, OA-11), cleaned with hot isopropanol (IPA) and air-dried before use, underwent surface modification by spin-coating a 1 wt%-IPA solution of a silane coupling agent (3-aminopropyltriethoxysilane (APTES, Shin-Etsu Silicone, Tokyo, Japan, KBM-903) and drying at 110 °C for 1 min on a hot plate. A set of PI films (length: 70 mm, width: 10 mm, and typical thickness: 20 μm, with 4 specimens in a row on each glass substrate) were compressed at room temperature onto the modified surface of the glass substrates at a fixed compression pressure of approximately 5 kgf/cm^2^ using a manual roll laminator. During this process, a 50 μm-thick PET film was interposed between the PI film and the laminator to prevent them from sticking together. The compression pressure was not further increased to prevent the glass substrates from collapsing. Laminating at lower compression pressures was also undesirable due to the risk of adhesion failure and data instability.

In this lamination process, a smoother surface of the homemade PI films (the surface facing the substrate in the PI films prepared using glass substrates) was always utilized to establish close and stable interfacial contact. The laminates were gradually heated to 150 °C and maintained at that temperature for 10 min in an air-convention oven. A commercially available super-heat-resistant PI film (TOYOBO, Osaka, Japan, XENOMAX_®_, thickness: 38 μm) was used for preparing a reference laminate sample.

The interfacial adhesion strengths (*σ*_ad_) of the laminates were estimated from the peel strengths (*σ*_peel_), which were measured by the 90° peel testing at a peeling rate of 100 mm/min using a peel tester. This apparatus comprises an automatic vertical servo stand/handy digital force gauge (Japan Instrumentation System, Sakurai-City, Nara-Prefecture, Japan, JSV-H1000), equipped with an automatic synchronous sliding stage to conduct the peel testing at a fixed angle of 90°, as shown in Figure 7b. A typical *σ*_peel_–peel interval curve is illustrated in Figure S4. The average peel strengths (*σ*_peel_) within a 50 mm peel interval were derived from the *σ*_peel_–peel interval curve using data analysis software (Japan Instrumentation System, SOP-PEEL).

#### 2.2.10. Contents of Imide and Fluorine Groups in the PI Structures

The contents (in wt%) of imide (O=C–N–C=O) group (*C*_i_) and fluorine (F) group (*C*_F_) in the PI structures were calculated to examine the correlations with *σ*_peel_ from the following equations:

*C*_i_ = [*F*_W_ (imide)/*F*_W_ (unit)] × 100 (6)

*C*_F_ = [*F*_W_ (F)/*F*_W_ (unit)] × 100 (7)

where *F*_W_ (imide), *F*_W_ (F), and *F*_W_ (unit) denote the formula weights of the imide, fluorine groups, and repeating units, respectively.

## 3. Results and Discussion

### 3.1. Difficulty in Simultaneously Achieving the Target Properties and Selection of Optimum Diamine for the Present Aim

**Figure 8:**
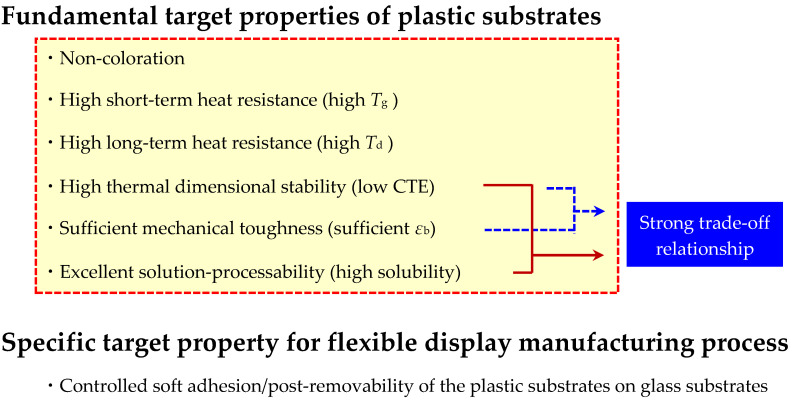
Fundamental and specific target properties of plastic substrates for flexible display applications.

Figure 8 illustrates the fundamental requirements for plastic substrates in flexible displays. In general, it is crucial to enhance the overall main chain linearity/rigidity of PIs to achieve reduced CTE properties [72,73]. However, this molecular design often leads to a notable decline in film toughness due to decreased chain entanglement. Therefore, a significant trade-off exists between a low CTE and high film toughness [74]. An increase in the chain linearity/rigidity also tends to cause a decrease in solubility (solution processability) owing to intensified chain aggregation [64], suggesting the difficulty of simultaneously achieving a low CTE and high solubility (Figure 8).

**Scheme 3:**
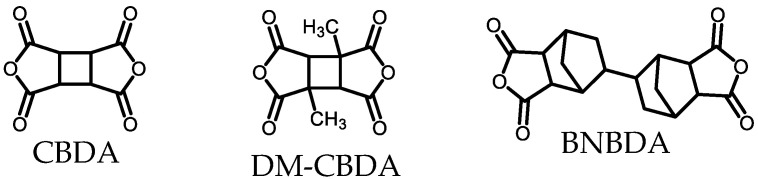
Molecular structures of CBDA, DM-CBDA, and BNBDA.

The main objective of this study is to achieve extremely high optical transparency, excellent heat resistance, low CTE, sufficient film toughness, and good solution processability simultaneously using semi-cycloaliphatic PI systems (B-type, Figure 1). However, cycloaliphatic TCDAs that may meet our goal are limited. For example, the range of available cycloaliphatic TCDAs that are effective in reducing CTE is limited to CBDA [48], its derivative (1,3-dimethyl-substituted CBDA (DM-CBDA) [41], and BNBDA [64] (Scheme 3).

Owing to the importance of the overall main chain linearity/rigidity for reducing the CTE, in addition to cycloaliphatic TCDAs, the aromatic diamines must have linear/rigid structures. However, the combination of rigid cycloaliphatic TCDAs with rigid aromatic diamines, such as the CBDA/*m*-TOL system (*m*-TOL: *m*-tolidine), renders the resulting PIs insoluble.

**Scheme 4:**
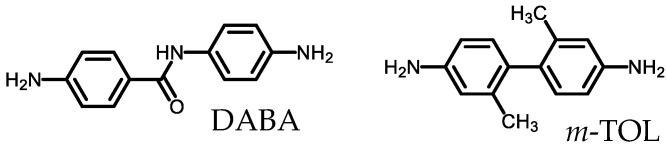
Molecular structures of DABA and *m*-TOL.

Furthermore, highly reactive and rigid aromatic diamines [typically, 4,4′-diaminobenzanilide (DABA) and *m*-TOL, Scheme 4] are often initially highly colored (especially DABA), owing to a colored impurity that is often difficult to separate. When utilized without pre-decoloring, the resulting PIs (even B-type) may not consistently yield completely colorless films.

Comprehensively considering the necessity of non-colored diamines along with the desired properties of complete transparency, low CTE, and solubility of resulting PIs, the most promising candidates for aromatic diamines for this purpose are virtually limited to TFMB. Unfortunately, the renowned CBDA/TFMB system lacks solution processability [41]. This study emphasizes CpODA and BzDAxx as crucial cycloaliphatic TCDAs and substitutes for CBDA.

### 3.2. Presumed Steric Structures and the Structural Linearity/Rigidity of CpODA and BzDAxx

The CpODA-based model compound (Document S1, Scheme S1) was recrystallized to acquire single crystals appropriate for X-ray structure analysis; however, this attempt was unsuccessful. Consequently, we inferred the steric structure of CpODA based on its synthetic pathway and the steric structures of the starting material and its intermediates.

Matsumoto et al. [75] utilized the *endo-endo* form-rich 5-norbornene-2-spiro-*α*-cyclopentanone-*α*′-spiro-2″-5″-norbornene (*endo-endo*-rich CpONE *trans*/*cis* mixture) as the starting material. They incorporated four methoxy carbonyl (COOMe) groups to the terminal C=C groups of CpONE in the presence of a catalyst (CuCl_2_/PdCl_2_), according to the method reported by Stille et al. [76]. The resultant norbornane-2-spiro-*α*-cyclopentanone-*α*′-spiro-2″-norbornene-5,5″,6,6″-tetracarboxylic acid tetramethyl ester (CpOME) was converted to the corresponding tetracarboxylic acid through transesterification with formic acid in the presence of *p*-toluenesulfonic acid anhydride. CpODA was obtained by dehydrating the tetracarboxylic acid at 170 °C and subsequent sublimation. In the final product, CpODA, the steric configuration of the starting material (CpONE) remained consistent across all reaction stages. Furthermore, it is established that the introduced COOMe groups have an all-*exo* configuration relative to the methylene head of the norbornane (NB) units [77].

**Figure 9:**
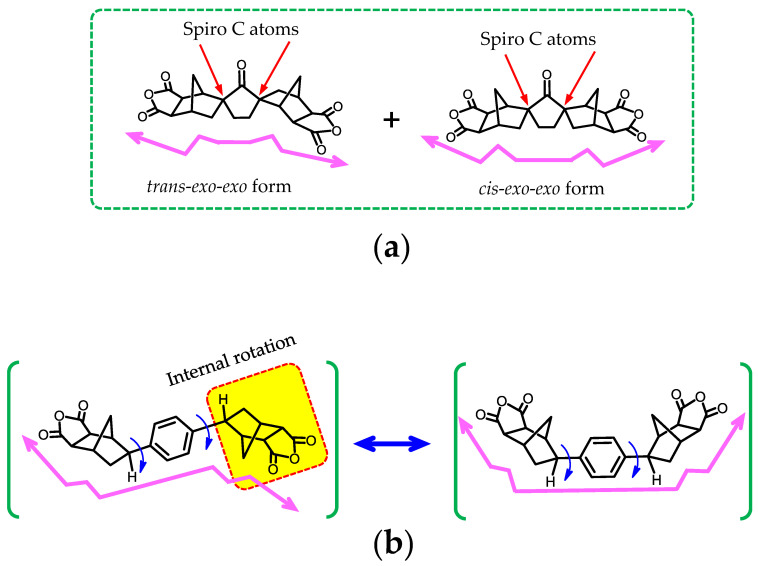
Presumed steric structures of the cycloaliphatic TCDAs used in this study: (**a**) *exo-exo*-rich CpODA *trans*/*cis* mixture obtained from *exo-exo*-rich CpONE *trans*/*cis* mixture [78] and (**b**) BzDAxx [79]. The inserted zigzag-arrows represent relative structural linearity/rigidity of these monomers.

Using a similar synthetic pathway, a different steric structure of CpODA (*exo-exo* form-rich CpODA, *trans*/*cis* isomer mixture) was commercially manufactured by ENEOS Corp. using a different steric structure of the starting material (*exo-exo* form-rich CpONE, *trans*/*cis* isomer mixture), as reported in the patent literature [78] (Figure S5). The steric structures and their mixture composition of the starting material, intermediates, and the final product (CpODA) were characterized using one- and two-dimensional ^1^H- and ^13^C-NMR spectroscopy and high-performance liquid chromatography (HPLC). The steric structure of the final product, *exo-exo*-rich CpODA, is depicted in Figure 9a. This final product (CpODA) has a unique rigid structure, where the central cyclopentanone unit is linked to the NB units on both sides through the spiro carbon atoms, around which internal rotation is prohibited. As indicated by the pink arrows in Figure 9a, these isomers (*trans-exo-exo* form and *cis-exo-exo* form) display considerable structural linearity. Therefore, the combination of *exo-exo*-rich CpODA and TFMB is more likely to yield a low-CTE PI film due to its main chain linearity. This is feasible provided that the polymerization led successfully to a very high *M*_w_ of PI, resulting in a high-quality ductile film.

**Scheme 5:**
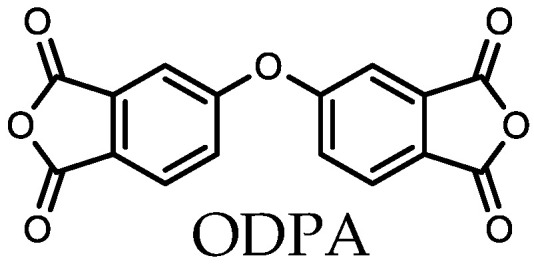
Molecular structure of ODPA.

The steric structure of BzDAxx was also inferred from its synthetic pathway and the steric structures of the starting material and intermediates (Figure S6) because our attempt to determine its steric structure by X-ray structure analysis using the corresponding model compound (Document S2, Scheme S2) was unsuccessful. BzDAxx was commercially produced by ENEOS Corp., as documented in the patent literature [79]. The steric structure of the starting material (*cis*-5-norbornene-*exo*-2,3-dicarboxylic anhydride (*exo*-NBDCA), specifically the all-*exo* configuration of the C=O groups, was maintained throughout all reaction steps. The second-step reaction between the C=C group of *cis*-5-norbornene-*exo*-2,3-dicarboxylic acid dimethyl ester (*exo*-NBDCME) and 1,4-dibromobenzene formed two NB–Ph bonds with an all-*exo* configuration, as confirmed by one- and two-dimensional ^1^H- and ^13^C-NMR spectroscopy [79]. As indicated by the pink-colored arrow lines in Figure 9b, BzDAxx maintains a certain level of structural linearity/rigidity compared to typical flexible monomers [e.g., 4,4′-oxydiphthalic anhydride (ODPA), Scheme 5], although its structural linearity somewhat diminishes due to the conformational change around the NB–Ph–NB bonds. Therefore, the combination of BzDAxx and TFMB, which is less structurally linear/rigid than the CpODA/TFMB system, is likely to exhibit a significantly lower CTE than common flexible PIs.

### 3.3. Applicability to Conventional Two-Step Process

#### 3.3.1. Polyaddition Reactivity of CpODA and BzDAxx with TFMB

Matsumoto et al. [50,51] conducted polyaddition of CpODA with common aromatic diamines and showed that the use of ether-containing diamines, such as 4,4′-ODA and 1,3-bis(4-aminophenoxy)benzene (TPER), produces flexible PI films through the conventional two-step process. However, the combination of CpODA and TFMB, which is of primary interest to us, has not been explored. Therefore, we first endeavored to produce a high-quality PI film by polyaddition of CpODA and TFMB and subsequent thermal imidization.

The results of the polyaddition of CpODA with aromatic diamines are summarized in Table 2. The CpODA/4,4′-ODA system (#1T) led to a homogeneous and viscous solution of PAA with an acceptable level of *η*_red_ (0.82 dL/g), slightly below an empirical threshold required for film formation (*η*_red_ > ~1 dL/g). Consistently, thermal imidization of the PAA cast film provided a self-supporting film without any cracks.

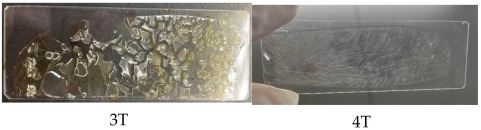



The CpODA/TFMB system (**#**2T) resulted in a homogeneous solution of PAA with an almost identical *η*_red_ value (0.83 dL/g) to that of CpODA/4,4′-ODA (#1T). Nonetheless, this system yielded a cracked PI film, indicating that the conventional two-step process was ineffective in producing flexible PI films for CpODA/TFMB.

**Figure 10:**
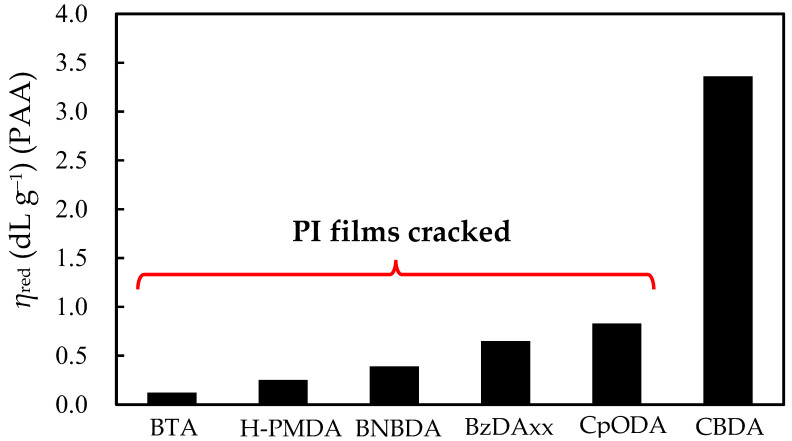
Comparison of the *η*_red_ (PAA)-based polyaddition reactivity of various cycloaliphatic TCDAs with TFMB. Polyaddition was conducted in DMAc at room temperature typically for 72 h at an initial monomer content of 30 or 40 wt%, during which the reaction mixture was gradually diluted with a minimal quantity of the same solvent to ensure effective magnetic stirring.

Figure 10 illustrates a comparison of the *η*_red_-based polyaddition reactivity of CpODA/TFMB (#2T) with its TFMB-based counterparts. H-PMDA and BTA exhibited very low *η*_red_ values (<0.3 dL/g), with their cast films containing numerous fine cracks [56]. The diminished polyaddition reactivity is probably attributed to self-steric hindrance from the spatially adjacent and all-*exo*-configuration functional groups in these TCDAs [40]. In contrast, BNBDA, BzDAxx, and CpODA showed discernibly improved *η*_red_-based polyaddition reactivity with TFMB, likely due to the spacer effect that minimizes the proximity of the functional groups during polyaddition. However, even these spacer-type TCDAs did not improve the film-forming ability.

In contrast, CBDA exhibited an outstandingly high *η*_red_ value and afforded a crack-free self-supporting PI film. This exceptionally high polyaddition reactivity is probably ascribed to ring strain in the functional groups connected to the four-membered central cyclobutane unit of CBDA [41]. The result of CBDA suggests that even in the currently focused CpODA/TFMB system, a flexible PI film is likely to become available if its *M*_w_ can be dramatically improved somehow. Unexpectedly, the different film preparation routes via chemical imidization provided a brittle but crack-free CpODA/TFMB PI film (#2C, Table 2), although the film flexibility was not sufficient for proper mechanical testing.

Table 2 presents the polyaddition reactivity of BzDAxx. The BzDAxx/4,4′-ODA system (#3T) resulted in a moderate *η*_red_ value of 0.62 dL/g, slightly lower than that of its CpODA-based counterpart (#1T), leading to a cracked PI film, in contrast to #1T, even though this level of *η*_red_ value is usually within the tolerance for film formation. The polyaddition of BzDAxx and TFMB (#4T) also yielded a moderate *η*_red_ value of 0.65 dL/g, yet resulted in a cracked PI film. Film preparation through chemical imidization (#4C) also did not improve the poor film-forming ability, unlike its CpODA-based counterpart (#2C). Consequently, neither the traditional two-step process nor the chemical imidization approach proved effective in producing a BzDAxx/TFMB PI film.

#### 3.3.2. Factors for Film Cracking

**Scheme 6:**
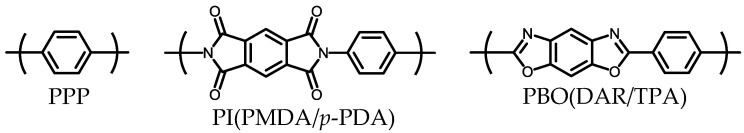
Typical polymers with rod-like main-chain structures.

**Figure 11:**
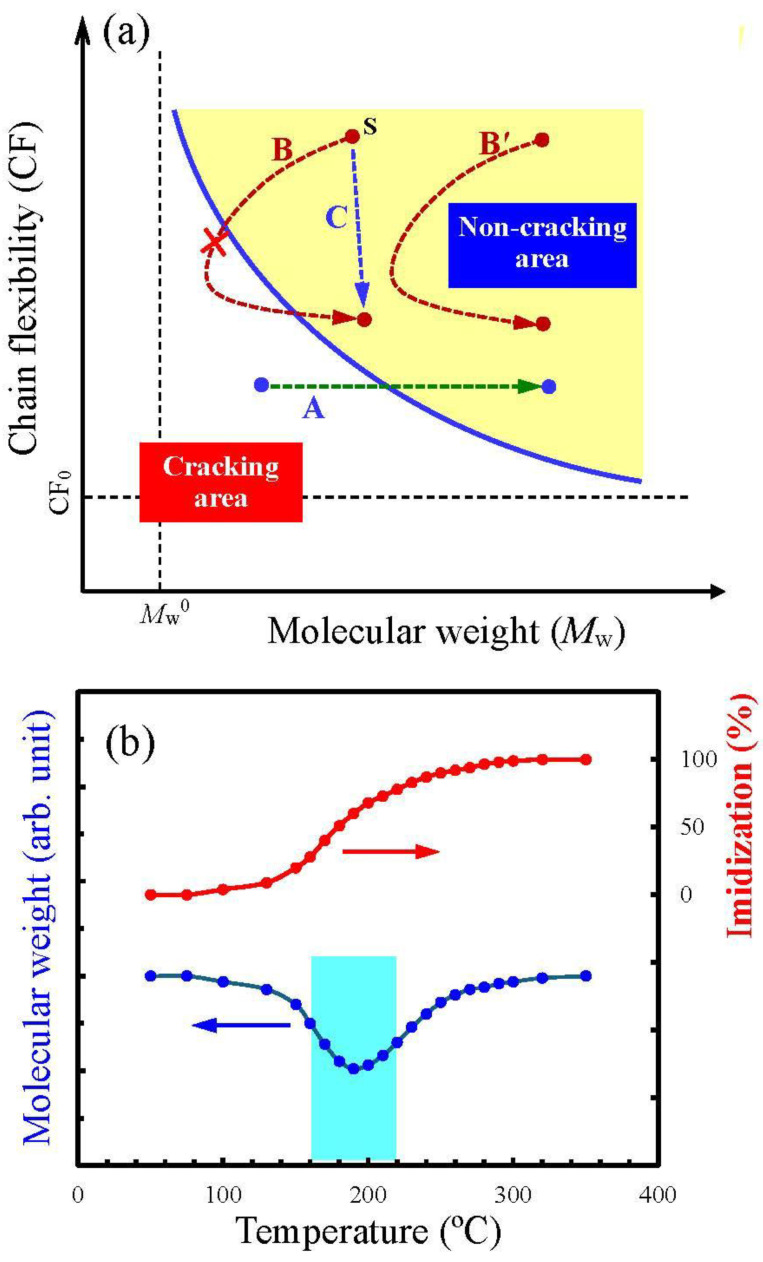
Schematic diagrams for understanding crack formation: (**a**) impact of the PAA/PI molecular weight (*M*_w_) and chain flexibility on the film-formability, and (**b**) changes in imidization (%) and *M*_w_ in the heating process. The inserted symbols are described in the text.

The distinct disparity in film-forming capability between #2T and #2C can be elucidated by the schematic diagram illustrating the relationship among polymer chain flexibility, *M*_w_, and film-formability (Figure 11a). Generally, enhancements in the rotational flexibility of polymer chains and *M*_w_ contribute to improved chain entanglement, thereby reducing crack formation [80]. Consequently, a hyperbolic-like curve (Figure 11a) can delineate the boundary between crack presence (unshaded area) and absence (yellow-shaded area), asymptotic to the lower-limit molecular weight (*M*_w_^0^) and lower-limit chain flexibility (CF_0_). Empirically, *M*_w_^0^ is approximately 10,000 or lower for common polymers (probably for flexible PIs as well), consistent with a prior finding [81]. For instance, in the PMDA/4,4′-ODA system, when the initial weight-average *M*_w_ of PAA dropped below approximately 10,000, the resulting PI film became significantly brittle. Conversely, entanglement-free virtual systems with CF_0_ resemble rod-like polymers such as poly(*p*-phenylene) (PPP), PI(PMDA/*p*-PDA) [82], and PBO(DAR/TPA) [83] (Scheme 6).

In general polymer systems, a common strategy to address severe embrittlement without altering their chemical compositions is simply to increase their *M*_w_s towards the non-cracking region (route A, Figure 11a). Conversely, during the thermal imidization of PAA cast films a complex transformation occurs along route B due to simultaneous alterations in the chain structure from PAA to PI and *M*_w_. The intricate *M*_w_ variation involves an appreciable decrease in *M*_w_ due to depolymerization in the medium temperature range (~170–220 °C) and subsequent recovery resulting from the recombination of temporarily formed functional groups at higher temperatures (*T* > 300 °C) [84,85,86], as shown in Figure 11b. When the initial *M*_w_ of PAA was relatively low, the film adhered to the substrate experiences cracking upon entering the cracked region (symbol **×**) during thermal imidization (route B). This cracking occurred when the film became intolerable due to a substantial reduction in *M*_w_ and the development of shrinkage stress, which was generated by the restraint of imidization-induced shrinkage by the substrate.

However, there is a possibility of preventing penetration into the film cracking area by shifting the start point S upward along route B (Figure 11a). This effect (increase in chain flexibility) corresponds to CpODA/4,4′-ODA (#1T) having film-forming ability, while CpODA/TFMB (#2T) does not. Additionally, a rightward shift of the starting point S, due to the increase in the initial *M*_w_ of PAA, is likely to keep it within the non-cracking area along route B’.

In contrast to the conventional two-step process, chemical imidization basically maintains the initial molecular weight of PAA throughout the reaction without depolymerization [30] (route C). This explains why #2C prevented film cracking, unlike #2T.

Thus, regardless of whether the two-step process or chemical imidization process is used, the key to producing flexible PI films is to dramatically enhance the initial *M*_w_s at the PAA stage. However, the attempt to further boost the *M*_w_s of PAAs has reached its limit. This is because polyaddition was carried out at extremely high monomer contents near the practical upper limit as the final effective approach.

### 3.4. Role of Modified One-Pot Polymerization Process

#### 3.4.1. Refinement of One-Pot Polymerization Conditions

The success of this study relies on the compatibility of the CpODA/TFMB and BzDAxx/TFMB systems with the modified one-pot polymerization process, leading to the production of PIs with extremely high *M*_w_s and resulting in highly tough PI films. The key requirement for the modified one-pot process is the exceptional solubility of the monomers and resulting PIs in the polymerization solvents. This enables the progress of the reaction at significantly high monomer contents while maintaining solution homogeneity throughout. Any occurrence of gelation or precipitation during the one-pot polymerization disrupts the subsequent film preparation process. Our modified one-pot polymerization process achieves its effects through the combination of the following factors;

(1) Solvents

**Scheme 7:**
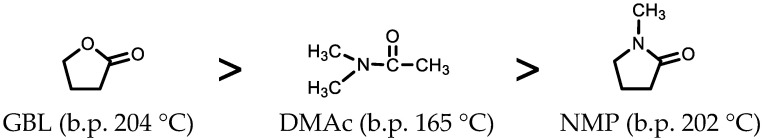
Superiority of the solvents in terms of suppressing the coloration of the reaction mixtures during one-pot polymerization.

Toxic phenolic solvents [87,88], which have been used in the conventional one-pot process, are absent in our modified one-pot process. Instead, we selected adequate aprotic organic solvents with sufficient dissolving capability, elevated boiling points favorable for full imidization, lower hygroscopicity, and minimized coloration during high-temperature reactions. Among these solvents, *γ*-butyrolactone (GBL) was deemed appropriate [56]. The empirical assessment reveals a diminishing trend in the superiority of polymerization solvents in terms of coloration suppression, as shown in Scheme 7.

However, there is a concern that GBL has lower dissolution capability than amide solvents. If ensuring the solution homogeneity during the one-pot polymerization is difficult, DMAc can be used. As illustrated in Figure 6, there were no issues with incomplete imidization, even when DMAc, with a lower boiling point, was employed. In cases where solution homogeneity was not preserved with DMAc, NMP was reluctantly used in this study.

(2) Azeotropic agents

Azeotropic agents (benzene derivatives such as toluene and xylene) have traditionally served as essential reagents to prevent the reverse reaction of imidization (hydrolysis of the formed PI) by promptly removing the by-produced water. However, in this study, these azeotropic agents were omitted to prevent a significant decrease in the original dissolution capability of the solvents due to the addition of these benzene derivatives with poor dissolution power. Indeed, the absence of azeotropic agents did not negatively impact the enhancement of *M*_w_, suggesting that the concerns regarding the hydrolysis of PI during the one-pot process are largely unwarranted.

(3) Temperature-rising profile

In the conventional one-pot polymerization process, polyaddition begins by adding TCDA solid to a diamine solution and stirring at room temperature for several hours. Subsequently, the resulting PAA solution is refluxed at elevated temperatures to complete the imidization. In contrast, our modified one-pot polymerization process excludes the above-mentioned first step. Instead, immediately after introducing TCDA to the diamine solution, the heterogeneous reaction mixture is swiftly heated to 200 °C with continuous mechanical stirring and maintained at this temperature for 4 h. This rapid heating method often proves more effective in increasing the *M*_w_ compared to the traditional temperature profiles [56].

(4) Reaction apparatus

In the traditional sealed reaction vessel, comprising a stirring blade, stirring rod, and through-hole-type Teflon mixing stopper with an inner O-ring, significant solvent/catalyst loss was inevitable during high-temperature one-pot polymerization. In contrast, a well-designed highly sealed reaction vessel, equipped with a mechanical stirrer with a non-contact magnetic coupling mechanism, was used in our modified one-pot polymerization process. This equipment efficiently minimized solvent and catalyst evaporation at 200 °C, thereby preventing gelation/precipitation and a decrease in the resultant *M*_w_s.

(5) Catalysts

In the traditional one-pot polymerization, less volatile and weakly basic catalysts, such as quinoline (b.p. 238 °C) and isoquinoline (b.p. 242 °C), have commonly been employed [89]. In contrast, a volatile and strongly basic compound, 1-ethylpiperidine (1-EP) (m.p. –8 °C, b.p. 131 °C), was used as the new imidization catalyst in our modified one-pot process [56]. Owing to the higher volatility of 1-EP, it became feasible to simply prepare the PI films through direct casting from the as-polymerized 1-EP-contaiing PI solution. Additionally, a less volatile and acidic cocatalyst, benzoic acid (BA, m.p. 124 °C, b.p. 249 °C), was utilized in this study. It was verified that BA could be readily eliminated during the isolation/washing process prior to solution casting.

**Figure 12:**
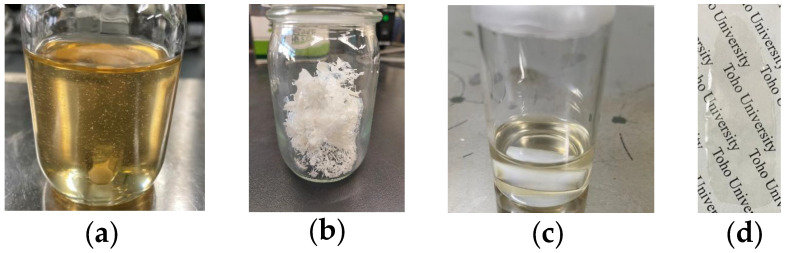
Appearance of a homogeneous PI solution obtained after modified one-pot polymerization (**a**), fibrous white powder form of the isolated PI (**b**), PI solution obtained by re-dissolution in fresh DMAc (**c**), and substrate-free PI film prepared via subsequent solution coating, drying, and annealing (**d**) for the CpODA/TFMB system.

Figure 12 exhibits the typical appearance at each stage in our modified one-pot process. The as-polymerized solution was somewhat colored due to the use of 1-EP (Figure 12a). This coloration was inconsequential as it could be readily eliminated during isolation and washing, as demonstrated by the white fibrous PI powder in Figure 12b. Re-dissolving this powder in a fresh solvent (DMAc or GBL) with a high solid content (typically, 15 wt%) yielded a non-colored and homogeneous PI solution (Figure 12c). Finally, colorless and non-turbid PI films were obtained via its coating, drying, and annealing (Figure 12d).

As the quantity of 1-EP increased, the *M*_W_s of the resultant PIs tended to rise gradually. However, the excessive use of 1-EP resulted in increased coloration of the solutions, potentially elevating the likelihood of a slight tint in the isolated PI powder. Therefore, our modified one-pot process was executed using an optimum amount of 1-EP (one equivalent to the theoretical dehydration amount during imidization).

Although the prominent *M*_w_ enhancement effect of the modified one-pot process resulted from a combination of refined items (1)–(5), the catalysts probably had the strongest impact.

#### 3.4.2. Impact of Imidization-Accelerating Catalyst on *M*_w_ Enhancement in the H-PMDA/TFMB System

**Figure 13:**
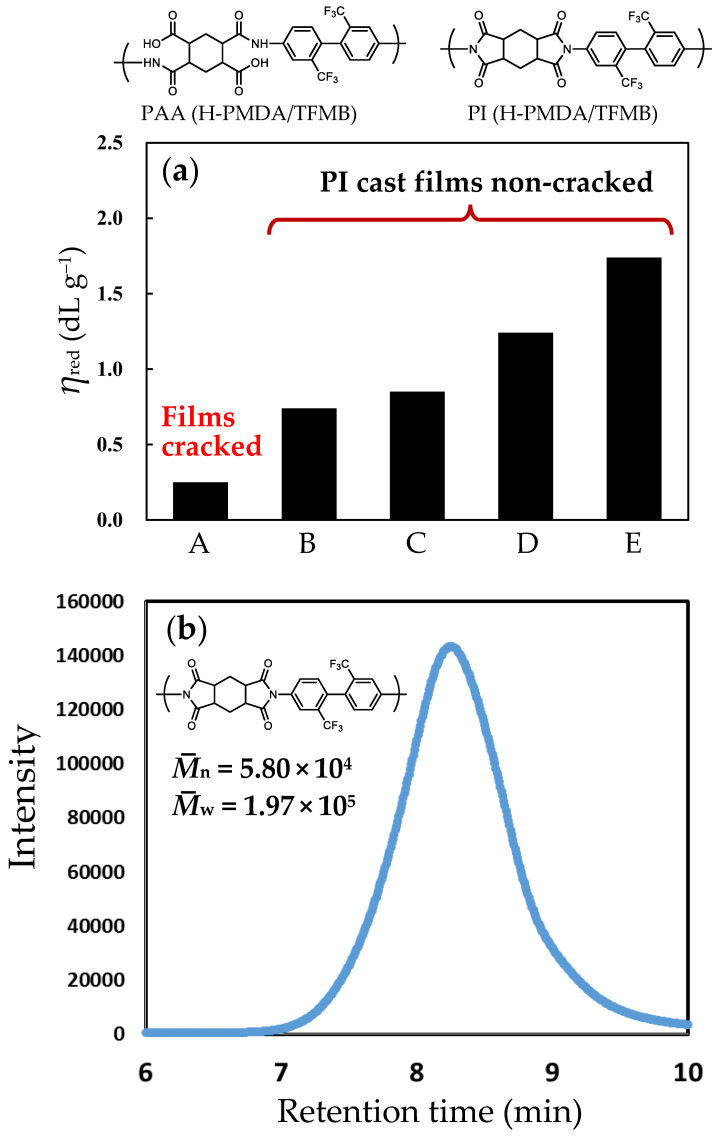
Effect of the polymerization process: (**a**) The *η*_red_ values and film-forming ability of the PAA or PI for the H-PMDA/TFMB system. (A) Polyaddition at room temperature in DMAc, (B–E) one-pot process in GBL, (B) no catalyst, (C) BA (1 equivalent (eq.)), (D) 1-EP (1 eq.), and (E) BA (1 eq.) + 1-EP (1 eq.), and (**b**) a typical GPC curve of H-PMDA/TFMB polyimide [condition (D)].

Figure 13a shows the effect of polymerization conditions on the *η*_red_ values of the resulting PAA or PI for the H-PMDA/TFMB system [56,64]. As previously mentioned, the polyaddition of H-PMDA and TFMB at room temperature resulted in a notably low *η*_red_ value, which was insufficient for film formation due to the relatively low reactivity of H-PMDA [Figure 13a, (A)]. Conversely, the one-pot polymerization without catalysts led to a slightly improved *η*_red_ value (B), enabling the formation of a crack-free PI cast film. The imidization-promoting effects of aromatic carboxylic acids such as benzoic acid (BA) and *p*-hydroxybenzoic acid have been suggested [90]. The addition of BA to this system showed only a limited effect (C). In contrast, the use of 1-EP (one equivalent) caused a prominent enhancement of the *η*_red_ value (D). This PI was found to have a high weight-average molecular weight (1.97 × 10^5^) from its GPC curve (Figure 13b). Unexpectedly, the combination of 1-EP (1 Eq.) and BP (1 Eq.) proved extremely effective in enhancing *η*_red_ (**E**). In comparison, polyaddition at room temperature was carried out in the presence of the same combined catalyst. However, no effect on *η*_red_ of the resultant PAA was observed.

Thus, even for PI systems without sufficient polyaddition reactivity and film-formability, our modified one-pot process using very effective catalysts has the potential to produce crack-free flexible films, as long as high solubility compatible with this process is ensured. The reason these imidization-accelerating catalysts exhibit the *M*_w_ enhancement effect is discussed later.

#### 3.4.3. Presumed Mechanism for the Effect of 1-EP/BA Combined Catalyst

**Figure 14:**
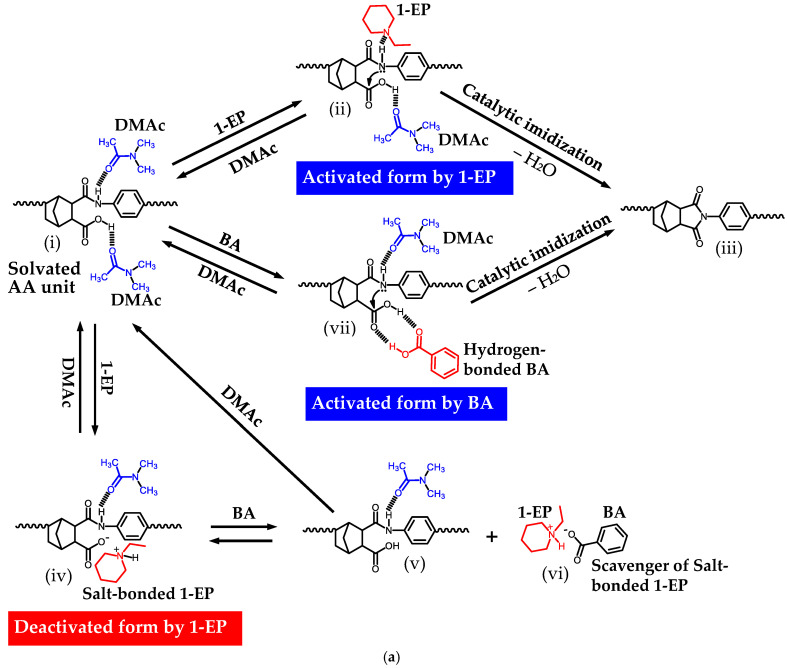

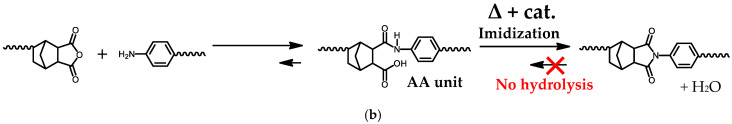
A reasonable mechanism for activating imidization by the 1-EP/BA combined catalyst in the modified one-pot process at an elevated temperature in solution (in DMAc as a typical solvent) (**a**) and the pseudo-one-way equilibrium for imidization of AA and ordinary equilibrium of the addition reaction between the terminal functional groups (**b**).

Figure 14a illustrates a reasonable mechanism for enhancing imidization reactivity using the 1-EP/BA combined catalyst. The COOH and NHCO groups in PAAs are solvated by forming hydrogen bonds with NMP [91,92], probably with DMAc as well [Figure 14a, form (i)]. Exchanging DMAc for 1-EP leads to the hydrogen-bonded (activated) form (ii), increasing the electron density on the nitrogen atom of the NHCO group. The heightened nucleophilicity to the carbonyl carbon atom of the COOH group accelerates the cyclodehydration towards the imidized form (iii). Conversely, the salt-bonded form (iv) between 1-EP and the COOH group results in an augmented electron density on the carbonyl carbon atom of the COOH group, leading to a decrease in the nucleophilicity of the NHCO nitrogen atom. Consequently, the salt-bonding leads to the deactivated form (iv) for imidization. Prolonged deactivation would nullify the overall catalytic impact of 1-EP. However, the neighboring BA can remove the salt-bonded 1-EP from the deactivated form (iv) as a “scavenger” and helps to restore its imidization reactivity through the solvated form (i) and the hydrogen-bonded form (ii) once more.

The hydrogen-bonded form (vii) between BA and the COOH group of the amide acid (AA) unit also contributes to an increase in imidization reactivity by reducing the electron density on the carbonyl carbon atom of the COOH group in the AA unit.

Thus, when 1-EP and BA are used together, the imidization reactivity can be maximized by combining the hydrogen-bonding effect of 1-EP with the scavenging effect of BA.

#### 3.4.4. Relationship Between Catalyst-Induced Imidization Acceleration and Molecular Weight Enhancement

Catalysts usually do not affect the equilibrium but solely boost the reaction rate (speed) [93]. Therefore, it is difficult to explain the reason why the imidization catalyst caused the *M*_w_ enhancement. To address this issue, we reconsider the equilibrium between AA and imide: AA ⇄ imide + H_2_O (Figure 14b). Assuming that the reverse reaction of imidization (i.e., hydrolysis of imide) is essentially insignificant due to the hydrolytic stability of PI and a limited water content in the reaction mixture at 200 °C even in the absence of azeotropic agents [64], imidization can be considered a pseudo-one-way reaction (AA → imide + H_2_O). With this reasonable premise, the catalyst-induced imidization acceleration results in the following secondary effects:

(1) A concomitant rapid decrease in the AA concentration ([AA]);
(2) A resultant rightward shift of the equilibrium of the addition reaction (acid anhydride + amine ⇄ AA).

The decrease in the concentration of the terminal functional groups (acid anhydrides and amines) corresponds to the following:

(3) Chain extension of PAA (= increase in *M*_w_ of PAA)
  = increase in the *M*_w_ of the PI.

Thus, the acceleration of imidization by the catalyst was closely linked to the *M*_w_ enhancement of the resultant PI. Accordingly, the prominent catalytic effect observed in Figure 13a is a direct outcome of this mechanism.

### 3.5. Polymerization Reactivity of CpODA and BzDAxx with TFMB in Modified One-Pot Process

The results of the modified one-pot polymerization of CpODA and aromatic diamines are summarized in Table 3. The CpODA/4,4′-ODA system (#1R) gave rise to immediate precipitation once the reaction mixture achieved homogeneity in hot NMP. The reaction mixture remained inhomogeneous even after stirring at 200 °C for 4 h (photograph at the bottom of Table 3) owing to the limited solubility of the resulting imide oligomer in NMP, as observed in other solvents (GBL and DMAc). This result contrasts with the fact that the modified one-pot polymerization of H-PMDA and 4,4′-ODA successfully proceeded in a homogeneous state even in GBL with a lower dissolution power [56].

The CpODA/TFMB system (#2R′) gave rise to partial gelation in GBL after the reaction mixture was once homogenized in the initial reaction stage, impeding subsequent film preparation, unlike the H-PMDA/TFMB reaction system in GBL (Figure 13a). The result of #2R′was very close to success. The *η*_red_ measurement of this system was feasible because the partially gelled reaction mixture became homogeneous after the significant dilution to 0.5 wt%. The high *η*_red_ value (3.29 dL/g) of #2R′ suggests that the polymerization was ongoing even in the partially gelled solution. The slightly insufficient solubility of CpODA/TFMB in GBL, causing the partial gelation, is related to the rotation-inhibited rigid spiro-structure of CpODA (Figure 9a). In contrast, the polymerization in DMAc with higher dissolution power (#2R) proceeded smoothly, resulting in the PI with an extremely high *η*_red_ (4.52 dL/g). In contrast, CBDA/TFMB proved incompatible with the one-pot process in all amide solvents, probably reflecting the greater structural rigidity of CBDA compared to CpODA.

The BzDAxx/4,4′-ODA system (#3R) also proved incompatible with the modified one-pot process, even in NMP, as evident from the resultant inhomogeneous reaction mixture (photograph at the bottom of Table 3).

**Scheme 8:**
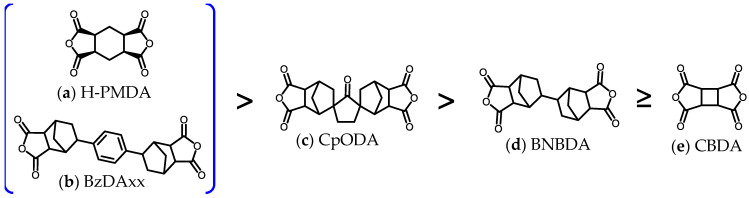
Superiority of the cycloaliphatic TCDA in terms of maintaining solution homogeneity during the one-pot polymerization process in lower-polarity solvents for the TFMB-based systems: (**a**) and (**b**) homogeneous in GBL, (**c**) inhomogeneous in GBL but homogeneous in DMAc, (**d**) inhomogeneous in GBL, and (**e**) inhomogeneous in amide solvents.

In the BzDAxx/TFMB system (#4R), the modified one-pot polymerization proceeded smoothly even in GBL, yielding a homogeneous/viscous solution of the PI with a very high *η*_red_ (4.00 dL/g). A comparison with CpODA/TFMB (#2R′), which was difficult to polymerize in GBL, suggests that BzDAxx offers greater advantages in ensuring the PI solubility compared to CpODA. This result is likely attributed to the relatively rigid yet rotation-allowed structure of BzDAxx (Figure 9b). In the modified one-pot polymerization using TFMB, the cycloaliphatic TCDAs exhibited decreasing superiority in achieving solution homogeneity in the following order (Scheme 8):

### 3.6. Properties of CpODA-Based PIs

#### 3.6.1. CpODA/4,4′-ODA System

Table 4 summarizes the properties of the CpODA-based PI films. The CpODA/4,4′-ODA system (#1T) produced a slightly colored PI film in appearance (YI = 9.5) using the conventional two-step process, as shown in the photograph below this table, although it still maintained a relatively high level of optical transparency. This slight coloration is possibly due to the inherent coloration of 4,4′-ODA and higher-temperature heat treatment needed for thermal imidization.

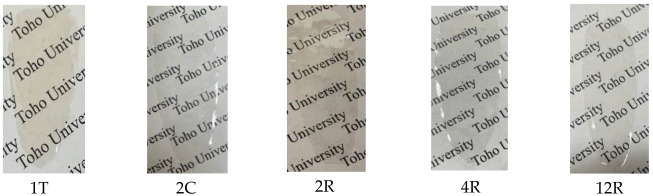



**Figure 15:**
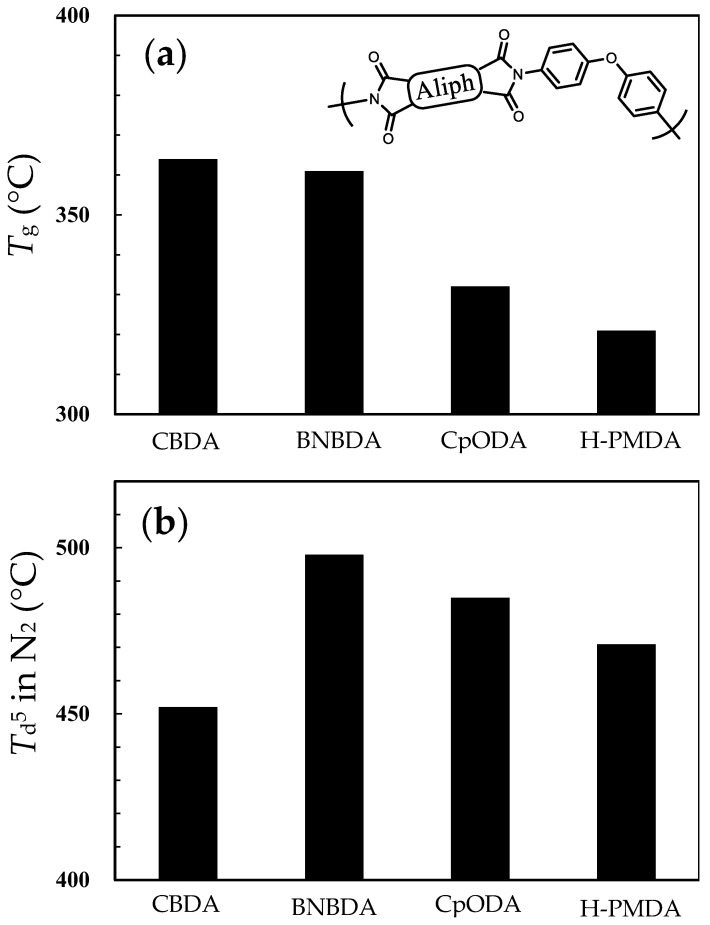
Impacts of cycloaliphatic TCDA on the heat resistance for the 4,4′-ODA-based PI films prepared via the two-step process: (**a**) *T*_g_ (by DMA) and (**b**) *T*_d_^5^ in N_2_.

The PI film (#1T) also exhibited a remarkably high *T*_g_ (332 °C), despite its high chain flexibility arising from the rotatable ether linkages in the main chain. This elevated *T*_g_ is probably ascribed to the rotation-inhibited rigid spiro-structure of CpODA (Figure 9a). When compared to its 4,4′-ODA-based counterparts (Figure 15a), the *T*_g_ of the PI films decreased depending on the cycloaliphatic TCDA structure in the order: CBDA > BNBDA > CpODA > H-PMDA. This sequence corresponds to the reverse order of the superiority of the one-pot process compatibility (i.e., solubility, Scheme 8), indicating a plausible relationship where solubility decreases with increasing *T*_g_.

Figure 15b shows the comparison of the thermal stability (*T*_d_^5^ in N_2_) of CpODA/4,4′-ODA (#1T) with those of its 4,4′-ODA-based counterparts. This PI (#1T) had a relatively high *T*_d_^5^ (485 °C), despite containing the thermally less stable cycloaliphatic unit in its structure. The *T*_d_^5^ (N_2_) value was obviously higher than those of its CBDA- and H-PMDA-based counterparts. This result is undoubtedly ascribable to the enhanced resistance of the bicyclo-structure of the norbornane (NB) unit in CpODA to fragmentation due to the C–C bond cleavage at elevated temperatures, compared with that of the monocyclo-structures [cyclohexane (CHx) and cyclobutane (CB) units]. The *T*_d_^5^ (N_2_) of CpODA/4,4′-ODA was somewhat lower than that of its BNBDA-based counterpart, probably owing to the presence of the monocyclo-cyclopentanone (Cp) central unit in the former. On the other hand, the CBDA/4,4′-ODA system exhibited a lower *T*_d_^5^ (N_2_) value than its H-PMDA-based counterpart, probably because of the presence of the monocyclo-CB unit, which includes ring strain, in the former. Consequently, the thermal stability of these 4,4′-ODA-based PIs decreased based on the local thermal stability of the cycloaliphatic structures in the following order: two bicyclo-NB (BNBDA) > two bicyclo-NB + monocyclo-Cp (CpODA) > single monocyclo-CHx (H-PMDA) > single monocyclo-CB (CBDA).

The PI film (#1T) did not show a low CTE (42.0 ppm/K), as anticipated with the use of flexible and non-linear 4,4′-ODA. However, this CTE was obviously lower than those of common polymers or highly flexible PIs (50–70 ppm/K [94]), probably reflecting the rigid and relatively linear structure of the CpODA-based diimide units in the PI chains (Figure 9a), which contributes to reducing the CTE.

While PI films obtained using 4,4′-ODA are often highly tough (typically, *ε*_b_ > 60% for PMDA/4,4′-ODA PI film [81]), the current PI film (#1T) showed much lower toughness (*ε*_b_^max^ = 15.9%) than anticipated, possibly due to its incomplete molecular weight [*η*_red_ (PAA) = 0.82 dL/g]. Therefore, enhancing its molecular weight significantly could potentially improve the film toughness. Unfortunately, this system (CpODA/4,4′-ODA) was incompatible with the promising modified one-pot process.

#### 3.6.2. CpODA/TFMB System

The properties of CpODA/TFMB film (#2C) prepared via chemical imidization are detailed in Table 4. The PI film exhibited excellent optically transparency with non-turbidity (*T*_400_ = 87.0%, YI = 1.7), as shown in the photograph below the table, and a low CTE of 23.7 ppm/K. However, despite its approximately sufficient *η*_red_ (0.99 dL/g), this film proved unexpectedly brittle, rendering it unsuitable for tensile testing.

In contrast, the modified one-pot process led to a dramatically improved *η*_red_ (4.52 dL/g) and acceptable film toughness (*ε*_b_^max^ = 16.3%), while maintaining very high transparency without turbidity (*T*_400_ = 85.0%, YI = 1.8, Haze = 0.94%, refer to the photograph below this table). The PI film (#2R) also exhibited a significantly low CTE (16.7 ppm/K), reflecting the linear/rigid structure of CpODA (Figure 9a). Note that this CTE value was even lower than that of the identical composition of PI (#2C). This is probably ascribed to the *M*_w_ effect on in-plane orientation induced during the casting of the PI solutions [42,64]. These findings underscore again the significance of maximizing the *M*_w_ of PIs.

**Figure 16:**
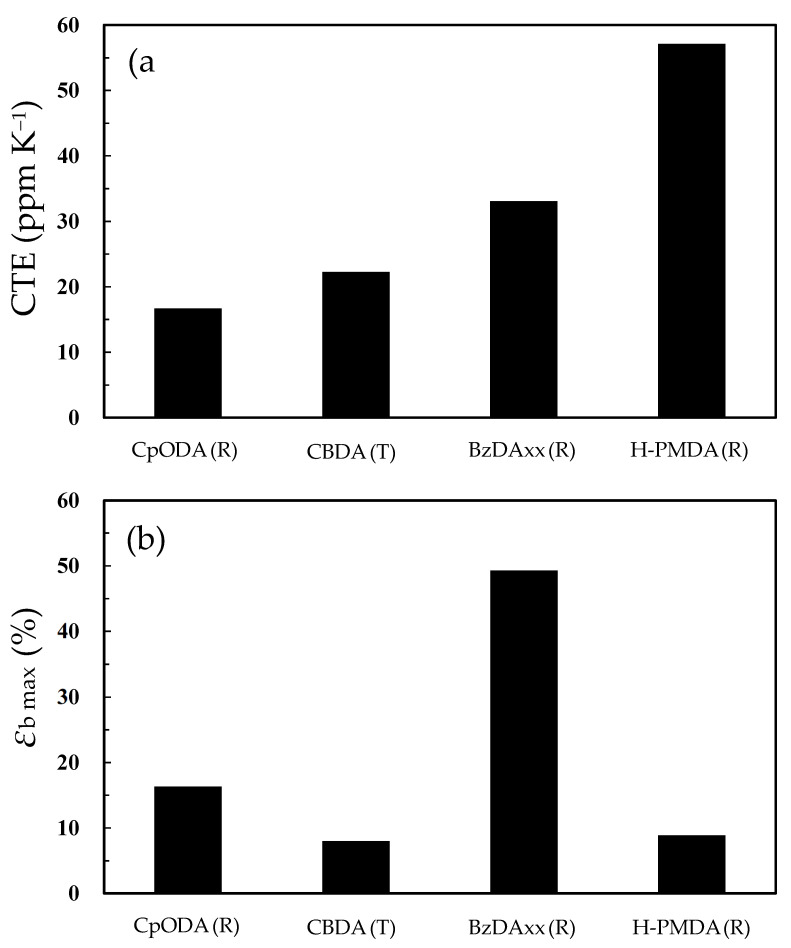
Impacts of cycloaliphatic TCDA on the selected properties for the TFMB-based PI films prepared via a modified one-pot polymerization (R) or two-step process (T): (**a**) CTE and (**b**) *ε*_b max_.

Figure 16a compares the CTEs of the TFMB-based related systems. The CTE of the CpODA/TFMB system (#2R) was lower than that of CBDA/TFMB (T), and this CTE was the lowest among the selected typical semi-cycloaliphatic PIs (B-type) using TFMB. The superiority of CpODA/TFMB is attributed to its maximized in-plane chain orientation due to the combined effect of its chain linearity/rigidity and the adoption of the film preparation route using homogeneous PI solutions [54]. Thus, this success was obtained by overcoming the trade-off between PI chain linearity/rigidity and solubility.

The film toughness for the TFMB-based related systems are compared in Figure 16b. The CpODA/TFMB system (#2R, *ε*_b max_ = 16.3%, champion data: *ε*_b max_ = 23.5%) has barely escaped from a category called “brittle film” (*ε*_b_ < 10%) (its typical examples: CBDA/TFMB and H-PMDA/TFMB). Thus, we recognized once more the difficulty in achieving high film toughness while ensuring high PI chain linearity/rigidity to significantly reduce the CTE.

**Figure 17:**
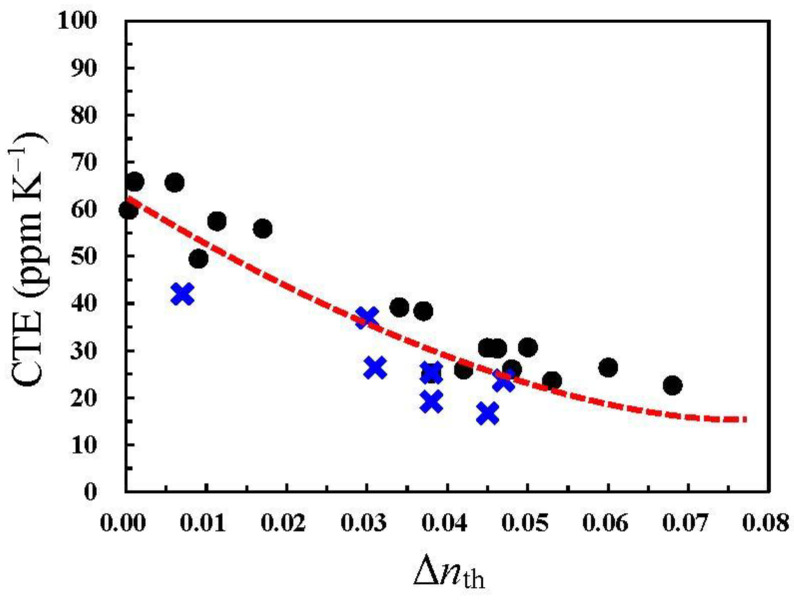
Correlation of the thickness-direction birefringence (Δ*n*_th_) and CTE for semi-cycloaliphatic PIs (B-type): (●) BNBDA-based PIs and related systems [64] and (**×**) the PI films examined in this study.

Figure 17 shows the Δ*n*_th_–CTE relationship, where the data of the present systems (B-type) were overlaid on the plots for the previously reported BNBDA-based and related systems (B-type) [64]. A certain degree of correlation, in which the CTE gradually decreases with increasing Δ*n*_th_, is observed in this figure. Therefore, the low CTE observed in CpODA/TFMB (#2R), as in other systems in this study, is not due to undesirable sample defective factors, such as residual strain, but an essential factor, *viz.*, the high extent of in-plane chain orientation [95], corresponding to the predicted chain linearity/rigidity.

The CpODA/TFMB system (#2R) also had an extremely high *T*_g_ exceeding 400 °C (Figures S2 and S3), reflecting its main-chain rigidity. Its relatively high thermal stability, as suggested by its *T*_d_^5^ (N_2_) value (478 °C), is related to the fragmentation-resistant bicyclo-structure of the NB units in CpODA. Moreover, it showed a very low optically estimated dielectric constant (*ε*_opt_ = 2.65), comparable to or slightly lower than that of CBDA/TFMB *ε*_opt_ = 2.66 [41]. This result is due to the combined effect of the absence of π-conjugation in the CpODA-based diimide local unit and the presence of two CF_3_ substituents with very low polarizability on TFMB. On the other hand, the water uptake of this film (#2R) was higher than expected (*W*_A_ = 1.1%), despite the presence of CF_3_ groups, and it was also much higher than that of the relevant system (*W*_A_ = 0.39% for CBDA/TFMB) [41]. This result may be due to the disturbed close chain stacking by the spiro-originated perpendicularly twisted steric structure of CpODA, which can act favorably for water penetration into the film.

Thus, the desired material simultaneously achieving excellent optical transparency, extremely high *T*_g_, and significantly low CTE was successfully obtained in this study, although there is room for further improvement in film toughness. This promising material could not be obtained without the modified one-pot polymerization method.

#### 3.6.3. Attempts to Improve the Film Toughness of CpODA/TFMB Using Common Flexible Diamines

We attempted to improve the film toughness of CpODA/TFMB while maintaining other excellent properties using a typical ether-containing comonomer, 4,4′-ODA, known for its effectiveness [56,74]. The modification of CpODA/TFMB by copolymerization with a minor content of 4,4′-ODA (10 mol%) (#5R) resulted in a slightly colored PI film (YI = 6.0) with a marginally reduced *T*_400_ value, despite the use of seemingly less colored 4,4′-ODA. The effect of this modification on the film toughness was certainly observed, although it was not so prominent. However, concomitantly, a slight increase in the CTE was unavoidable. A further increase in the 4,4′-ODA content could potentially improve the film toughness more significantly. However, this modification using 4,4′-ODA was halted due to the anticipated adverse effects on the optical transparency and low CTE property.

The toughening effect was also investigated using BAPP, which is empirically one of the most effective diamines for improving PI film toughness [64,65]. Unexpectedly, the modification of CpODA/TFMB using BAPP (30 mol%) (#6R) virtually caused no discernible deterioration of the optical transparency (YI = 1.3), in contrast to the afore-mentioned approach using 4,4′-ODA. The copolymer film (#6R) also exhibited clearly improved toughness (*ε*_b max_ = 34.1%) and still maintained a relatively low CTE (26.3 ppm K^−1^) and an extremely high *T*_g_ (362 °C), despite the use of highly flexible BAPP (30 mol%). Therefore, BAPP is an effective toughness modifier.

### 3.7. Properties of BzDAxx-Based PIs

The properties of BzDAxx/TFMB (#4R) are detailed in Table 4. The modified one-pot process dramatically enhanced the *M*_w_ of this PI and facilitated the formation of flexible PI films. The BzDAxx/TFMB system (#4R) generated a colorless and non-turbid PI film (*T*_400_ = 86.5%, YI = 1.4, Haze = 1.14%), as shown in the image at the bottom of this table. The YI value was lower than that of the CpODA/TFMB film (#2R, YI = 1.8) cast from the DMAc solution, probably owing to the use of GBL for solution casting in the former, which has greater resistance to discoloration during heating than DMAc (Scheme 7).

The BzDAxx/TFMB system (#4R) showed a significantly high *T*_g_ (372 °C), reflecting the relatively rigid structure of BzDAxx (Figure 9b), although the *T*_g_ was somewhat lower than that of its CpODA-based counterpart (#2R) (*T*_g_ = 411 °C). The PI film (#4R) also exhibited an intermediate CTE (33.1 ppm/K), positioned between those of CpODA/TFMB (CTE = 16.7 ppm/K) and common flexible PIs (50–70 ppm/K) such as H-PMDA/TFMB, as illustrated in Figure 16a. These findings do not conflict with our presumption that BzDAxx has somewhat lower structural linearity/rigidity than CpODA (Figure 9). Incidentally, the CTE of #4R was similar to that of the thermally imidized *s*-BPDA/TFMB film (CTE = 33.8 ppm/K) [96] (*s*-BPDA = 3,3′4,4′-biphenyltetracarboxylic dianhydride), which was obtained using a typical rigid aromatic TCDA, *s*-BPDA.

The film toughness of BzDAxx/TFMB (#4R) was compared with that of its TFMB-based counterparts in Figure 16b. It is notable that this PI film (#4R) exhibited remarkably high film toughness (*ε*_b max_ = 49.3%), despite the absence of rotatable ether linkages in the chain structure. The significant toughening mechanism of BzDAxx remains unclear. Nonetheless, BzDAxx is anticipated to be a new type of promising toughness modifier, distinct from traditional ones (e.g., 4,4′-ODA) that inevitably cause a significant increase in CTE due to their flexible ether linkages.

Our initial prediction was that BzDAxx/TFMB would exhibit higher thermal stability [*T*_d_^5^ (N_2_)] than CpODA/TFMB, owing to the presence of the thermally stable central 1,4-phenylene unit in BzDAxx. However, in fact, BzDAxx/TFMB showed a comparable or slightly lower *T*_d_^5^ (N_2_) (472 °C) than that of CpODA/TFMB (478 °C). This observation suggests that the C_4_–C_5_, C_5_–C_6_, and C_5_–H bonds in the NB unit of BzDAxx may be relatively susceptible to bond cleavage, resulting in the generation of relatively stable benzyl radicals at high temperatures.

### 3.8. Modification of CpODA/TFMB Using BzDAxx

The properties of the CpODA;BzDAxx/TFMB copolymers with varying BzDAxx contents are summarized in Table 4. Copolymerization with BzDAxx led to somewhat reduced *T*_g_s, compared to that of the pristine CpODA/TFMB system (#2R). Nonetheless, an extremely high *T*_g_ (383 °C by DMA) was maintained even at a high BzDAxx content of 50 mol%. The *T*_d_^5^ (N_2_) ranged from 360–370 °C in the BzDAxx content range of 10–50 mol% with neither a trend of an increase nor decrease in *T*_d_^5^ (N_2_) with varying BzDAxx content.

**Figure 18:**
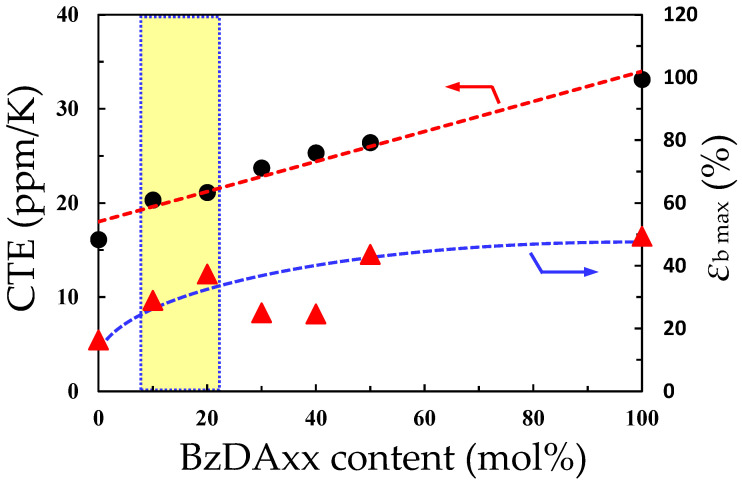
Impacts of the CpODA content on the CTE and *ε*_b max_ of the CpODA;BzDAxx/TFMB copolymers.

An increase in the BzDAxx content caused a gradual increase in the CTE. However, maintaining sufficiently low CTEs was possible at minor BzDAxx contents [e.g., CTE = 21.1 ppm/K at BzDAxx = 20 mol% (#9R)]. Figure 18 exhibits the dependence of the CTE on the BzDAxx content, showing a good linear relationship. Achieving a balance between low CTE and high film toughness was successful at BzDAxx contents ranging from 10 to 20 mol% (highlighted in yellow in Figure 18).

**Figure 19:**
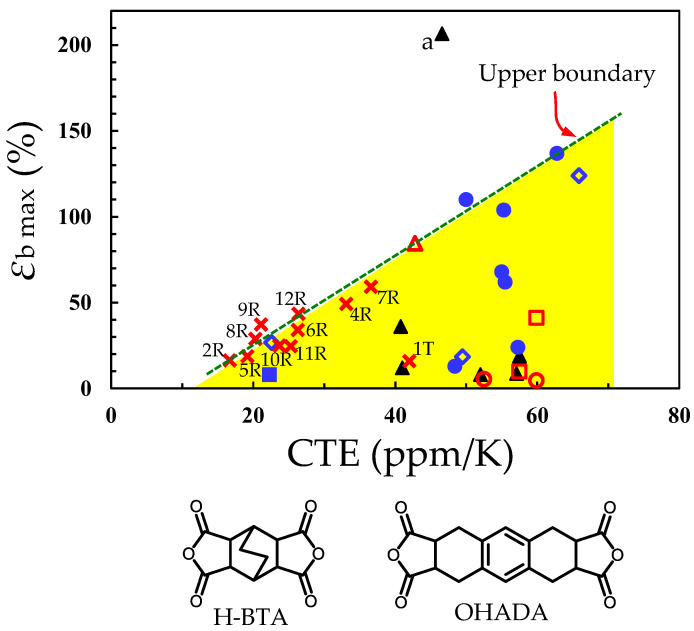
Correlation of CTE and *ε*_b max_ for the semi-cycloaliphatic PIs (B-type): (**◊**) BNBDA-based systems [64], (■) CBDA/TFMB, (▲) H-PMDA-based systems, a: H-PMDA/4,4′-ODA [56], (●) H-BTA-based systems [65], (○) OHADA-based systems [58], (□) H-BPDA systems [64], and (×) CpODA- and BzDAxx-based systems (this study). A typical conventional wholly aromatic PI (PMDA/4,4′-ODA) (Δ) [96] is also plotted for comparison.

As discussed in Section 3.1 and Figure 8, Figure 19 specifically represents the difficulty in simultaneously achieving a low CTE and high film toughness. Interestingly, a clear “upper boundary” curve, which is very difficult to go over upward, emerged in the CTE–*ε*_b max_ plot for various semi-cycloaliphatic PIs (B-type), with one exception of the H-PMDA/4,4′-ODA system (symbol *a* in Figure 19), of which the outstanding toughening mechanism is not well understood [65]. One notices that most of the plots for the CpODA- and BzDAxx-based PIs and related systems examined in this study are positioned in the vicinity of the upper boundary or beyond it (#8R and #9R). These are rare cases that simultaneously achieved a low CTE and high film toughness.

### 3.9. Compatibility with the Controlled Soft Adhesion/Easy Removal Process

#### 3.9.1. Impact of PI Structure on the Adhesion Strength

**Figure 20:**
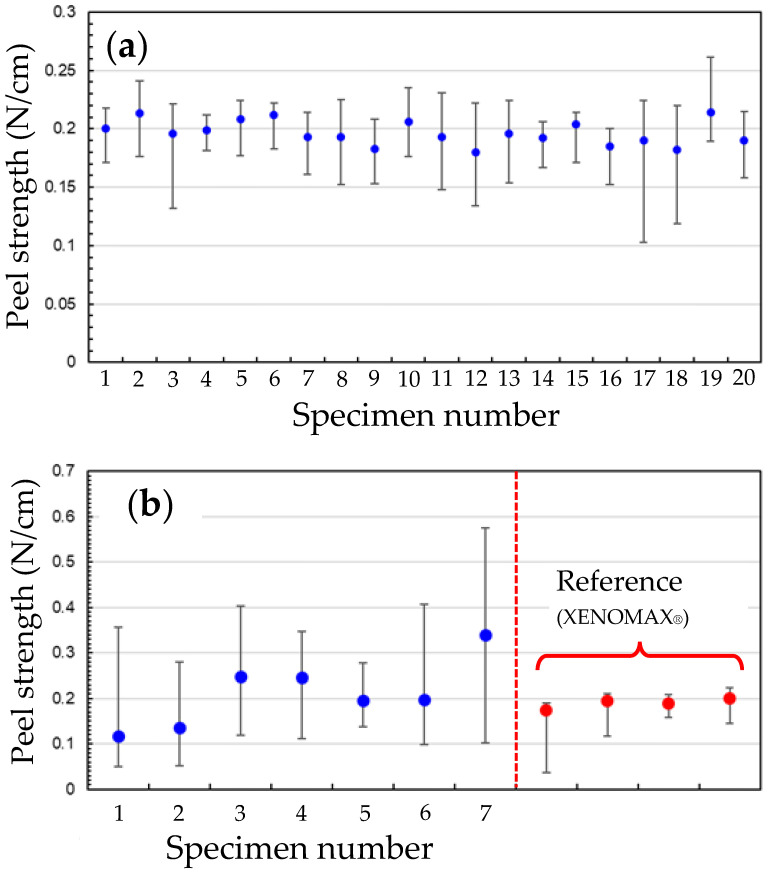
Peel strength and its distribution for the laminate samples of glass substrate and PI films: (**a**) XENOMAX_®_ film and (**b**) CpODA(90);BzDAxx(10)/TFMB copolyimide film. The symbols (●) and the error bars denote the average and minimum/maximum values during each peel testing.

Figure 20a displays the results of the 90° peel testing for the laminates comprising XENOMAX_®_ film and the surface-modified glass substrate as a reference. Interfacial delamination consistently occurs during peel testing. The reference laminate samples showed an appropriate peel strength (*σ*_peel_ = 0.20 N/cm) for the controlled soft adhesion/easy peel process with minimal data scattering, attributed to the excellent surface flatness of XENOMAX_®_ film and its high homogeneity [97]. Similarly, the adhesion strengths of the laminate samples using laboratory-made PI films were evaluated, as typically shown in Figure 20b, although an appreciable increase in data dispersion was unavoidable. The reference samples were concurrently prepared with the laminate samples using laboratory-made PI films to validate whether the reference samples maintained their original *σ*_peel_ values in each measurement.

**Figure 21:**
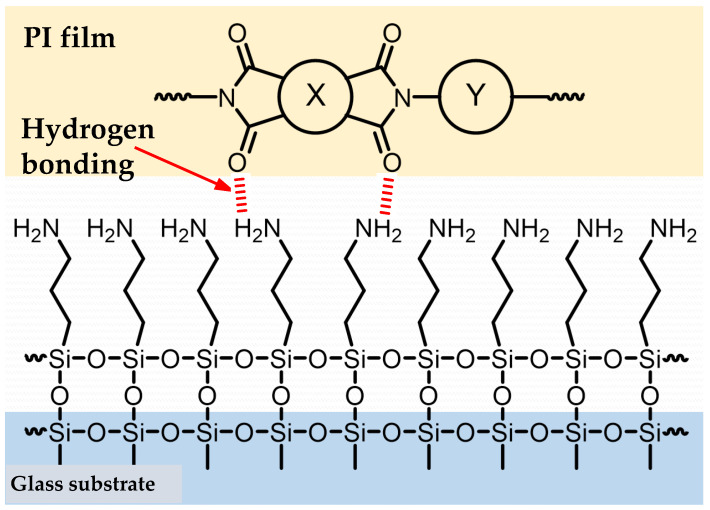
A presumed adhesion-improving mechanism by APTES for the conventional laminates prepared from the surface-modified glass substrates (solid) and PAA/PI solutions (liquid), and the mild pressure-induced solid–solid lamination between the PI films and APTES-modified glass substrates.

A common method to improve the adhesion between the PI layer and inorganic substrates (copper, silicon, glass substrates, etc.) involves by their surface modification using silane coupling agents such as APTES, as extensively documented [98]. On APTES-treated glass substrates, the APTES layer is fixed to the glass surface through three-dimensional crosslinks formed by the reaction between the triethoxy groups of APTES and the silanol groups on the glass surface. Note that the PI layer is usually formed by coating fluid solutions of PI or its precursor onto the APTES-modified glass substrate. In addition, it is well-known that aliphatic amino groups have a preference for hydrogen bonding with the imide C=O groups of PIs [99]. Considering these findings comprehensively, the improved adhesion using APTES in the traditional laminates likely arises from the mechanism illustrated in Figure 21.

The results of Figure 20 can probably be ascribed to a similar mechanism. However, a query persists, as this solid–solid lamination presents a significantly restricted opportunity for intimate molecular contact at the interface, in contrast to the traditional lamination employing polymer solutions. This study aimed to experimentally identify a specific PI structural factor (if any) governing adhesion strength, rather than elucidating the solid–solid adhesion mechanism.

Table 5 summarizes the adhesion strengths of the solid–solid laminates using various transparent PI films and their suitability for the controlled soft adhesion/easy peel process (rank 5 indicates the highest compatibility, as explained later). The majority of the colorless PI films examined in this study were incompatible with the controlled soft adhesion/easy peel process, as evident from their significant upward/downward deviations from the optimal peel strength range (*σ*_peel_ = 0.15–0.30 N/cm).

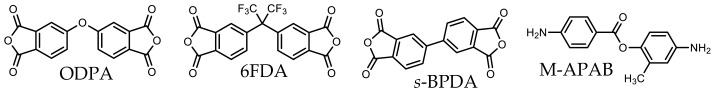



**Scheme 9:**
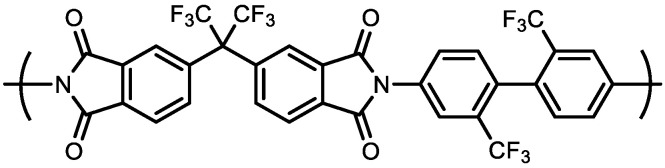
Structure of 6FDA/TFMB polyimide.

To discover a PI structural factor by which *σ*_peel_ can be intentionally controlled, we first explored the impact of the imide group content (*C*_i_) on *σ*_peel_ due to its potential role in hydrogen bonding, as illustrated in Figure 21. However, no clear correlation was found between *C*_i_ and *σ*_peel_, as demonstrated in Figure S7a. We further assessed the influence of introducing fluorine groups on *σ*_peel_ using a highly fluorinated 6FDA/TFMB polyimide film (#15C, Scheme 9). It was anticipated that the resulting laminate would show a notably low *σ*_peel_, according to the general understanding that fluorine groups typically compromise adhesion. Surprisingly, the solid–solid laminate constructed with 6FDA/TFMB PI film displayed a rather high *σ*_peel_ (1.68 N/cm), which was deemed unsuitable for the intended application. Indeed, there was no discernible correlation between the fluorine content (*C*_F_) and *σ*_peel_ (Figure S7b).

**Scheme 10:**
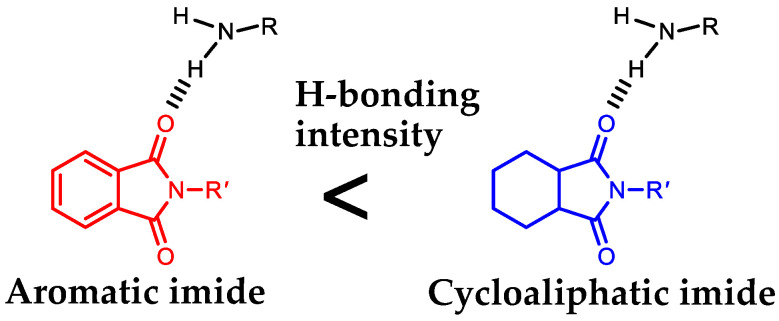
Relative intensities of hydrogen bonding between aliphatic NH_2_ and imide C=O groups.

The impact of aromaticity of the imide unit in the PI films on *σ*_peel_ was also investigated. It is widely acknowledged that aromatic imide compounds exhibit a lower electron density on the oxygen atom of the imide C=O group due to the partial delocalization of lone-pair electrons on the oxygen atom to the π-conjugated aromatic unit, compared to its cycloaliphatic imide counterparts lacking such delocalization. Therefore, the latter can establish stronger hydrogen bonds with the aliphatic NH_2_ groups than the former (Scheme 10).

**Scheme 11:**
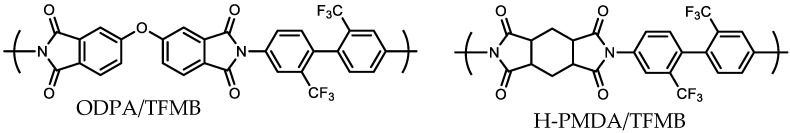
Structures of ODPA/TFMB and H-PMDA/TFMB polyimides.

According to this analysis, it is predicted that the solid–solid laminate using wholly aromatic ODPA/TFMB PI film (#14T, Scheme 11) will exhibit an obviously lower *σ*_peel_ than its counterpart using semi-cycloaliphatic H-PMDA/TFMB PI film (#13R, Scheme 11). However, in fact, the former showed a comparable *σ*_peel_ (3.41 N/cm) to that of the latter (3.58 N/cm), in contrast to our initial prediction.

On the other hand, contrary to our initial prediction, the laminate using *s*-BPDA/TFMB (#16T) showed a significantly reduced *σ*_peel_ (0.14 N/cm), compared to that using ODPA/TFMB (#14T), even though these are the same type of PIs, consisting of aromatic TCDAs and TFMB.

**Scheme 12:**
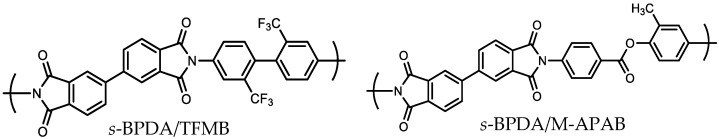
Structures of *s*-BPDA/TFMB and *s*-BPDA/M-APAB.

We also observed another unexpected result, in that the use of *s*-BPDA/M-APAB PI film (#17T, Scheme 12) [100] led to a rather low *σ*_peel_ (0.13 N/cm), close to that of partially fluorinated *s*-BPDA/TFMB (#16T, Scheme 12), despite the absence of the adhesion-hindering fluorine groups in the structure of the former.

Thus, no correlation was observed between *σ*_peel_ and the PI structural parameters examined here (*C*_i,_ *C*_F_, and the aromaticity of the imide units). Therefore, it was unsuccessful to find an effective chemical approach to appropriately adjust the adhesion strength of the solid–solid laminates.

#### 3.9.2. A Possible Hidden Parameter Dominating the Adhesion Strength

As previously mentioned, the mild-pressure-induced solid–solid lamination illustrated in Figure 20 was not always readily understood because of the limited opportunity for close molecular contact at the interface. However, assuming that the PI chains on the top surface, as well as the APTES layer formed on the glass surface, have “unfrozen” molecular motion, the observed solid–solid adhesion becomes understandable. In relation to this concept, a previous study [101] demonstrated that a common polymer film has a rather low *T*_g_ near the surface in comparison to its bulk *T*_g_, suggesting the presence of polymer chains with increased mobility near the surface. In addition, it is well-established that the rubbing treatment of PI films causes the chain alignment in the rubbing direction near the surface, leading to liquid crystal alignment through interaction with the rubbed PI surface [102]. This rubbing effect is also probably closely associated with the “soft” surface.

**Figure 22:**
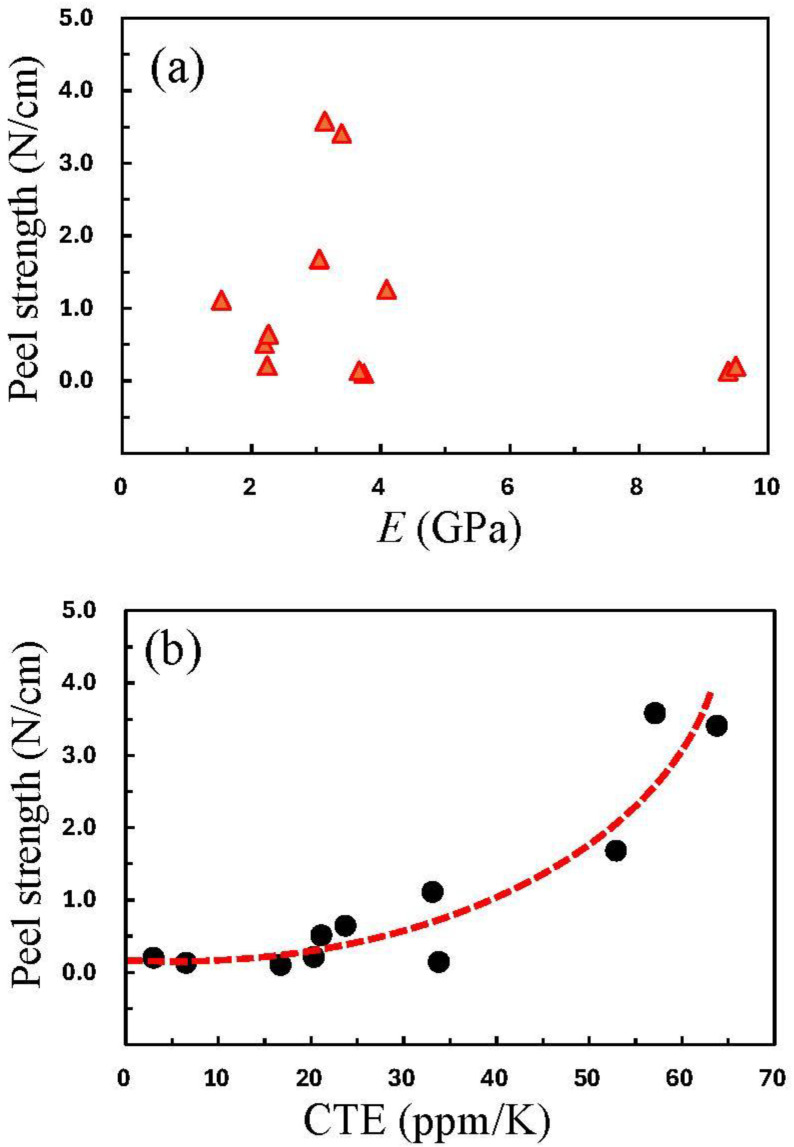
Correlation of the average peel strength and parameters relating to molecular rigidity for the semi-cycloaliphatic and wholly aromatic PI systems listed in Table 5: (**a**) tensile modulus (*E*) and (**b**) CTE.

Based on this concept, it can be assumed that the solid–solid adhesion is also closely related to the “surface mobility”, which should depend on the PI chain structures. Then, we first examined the correlation between *σ*_peel_ and the tensile modulus (*E*), which is an experimentally available parameter associated with bulk chain rigidity. Assuming that bulk chain rigidity is in a parallel relationship with surface rigidity, *σ*_peel_ may decrease with an increase in *E* owing to an increase in surface rigidity. However, no correlation was found in the *σ*_peel_–*E* plot, as shown in Figure 22a. Another bulk parameter, CTE, can be related to surface rigidity based on an empirical rule that rigid/linear PI chain structures contribute to a decrease in CTE [72,73,96]. Interestingly, a good correlation was observed between *σ*_peel_ and CTE, as shown in Figure 22b; *σ*_peel_ monotonously increased with increasing CTE, likely due to increased surface mobility. This result suggests that optimizing the adhesion strength of solid–solid laminates can be achieved using CTE as an indicator rather than the chemical factors based on the PI structures. Specifically, to adjust *σ*_peel_ to the optimum range (0.15–0.3 N/cm), it appears that the CTE needs to be approximately 20 ppm/K.

#### 3.9.3. Attempt of Simultaneous Control of CTE, Film Toughness, and Adhesion Strength

**Figure 23:**
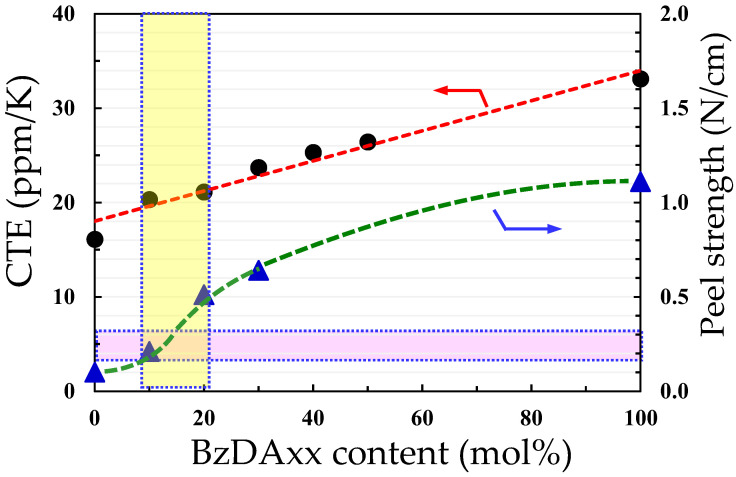
Impacts of the BzDAxx content in the CpODA;BzDAxx/TFMB copolyimides on the peel strength (*σ*_peel_) for the laminates of the copolyimide films and surface-modified glass substrates. The CTE data for the copolyimides are also re-plotted in this figure. The pink-shaded area represents the optimal *σ*_peel_ range for the soft-adhesion/easy removal process. The yellow-shaded area shows the optimal BzDAxx content range in the copolyimides for achieving both low CTE and sufficient film toughness.

Considering the above results comprehensively, a promising strategy to simultaneously achieve a low CTE, high film toughness, and appropriate adhesion strength of the laminates is only modifying CpODA/TFMB (#2R) by copolymerization with an adequate content of BzDAxx. The results of this approach are illustrated in Figure 23. The pristine CpODA/TFMB (#2R) (BzDAxx content = 0 mol%) exhibited a slightly lower *σ*_peel_ (0.10 N/cm) than the optimal range (0.15–0.3 N/cm), indicated by a pink-shaded region in this figure. The *σ*_peel_ monotonously increased with an increase in BzDAxx content. In addition, as detailed in Section 3.8 and Figure 18 the copolymer composition for simultaneous maintaining low CTE and sufficient film toughness ranged from 10–20 mol% of the BzDAxx content (highlighted in yellow). Therefore, the desired properties can only be met within a narrow range, where these two highlighted regions coincide, approximately at BzDAxx = 10 mol%.

### 3.10. Performance Balance

To practically apply heat-resistant colorless polymeric materials to plastic substrates in flexible displays, it is essential not only to achieve individual required properties at a high level but also to maintain their balance. In this study, the performance balance of the typical PIs investigated was visually assessed using a spider chart. The achievement level of each target was rated on a five-grade scale, following the assessment criteria outlined in Table 6. These criteria were objectively established based on the existing maximum and minimum values of the individual required properties, as detailed in our previous study [64]. Additionally, the compatibility of the solid–solid laminates using our PI films with the controlled soft adhesion/easy peel process was included in the spider charts, although this evaluation was three-grade; the laminates exhibiting the optimal range of *σ*_peel_ (0.15–0.30 N/cm) were assigned a rank of 5. On the other hand, the laminates with a completely inapplicable *σ*_peel_ range (*σ*_peel_ > 1 N/cm or *σ*_peel_ < 0.01 N/cm) were ranked 1. The medium *σ*_peel_ range (0.05–0.08 N/cm or 0.4–0.6 N/cm), suggesting that there still remains the potential for this application, was assigned to rank 3.

**Figure 24:**
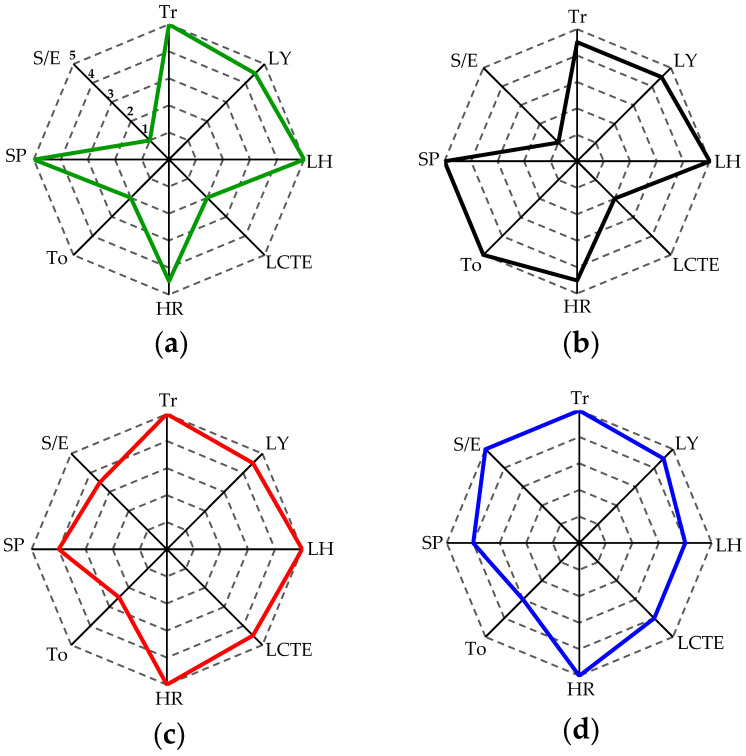
Performance balance of CpODA/TFMB and related systems: (**a**) H-PMDA/TFMB, (**b**) 6FDA/TFMB, (**c**) CpODA/TFMB, and (**d**) CpODA(90);BzDAxx(10)/TFMB. The ranking criteria of the target properties and the inserted abbreviations are shown in Table 6.

Figure 24 displays the spider charts that provide an overview of the performance balance of the selected PIs. A conventional semi-cycloaliphatic PI (B-type), the H-PMDA/TFMB system (#13R), exhibited significant imbalance due to multiple unachieved items, evident from the notably concave spider chart in Figure 24a. In the wholly aromatic 6FDA/TFMB system (#15C) (Figure 24b), film toughness, which was one of the drawbacks in H-PMDA/TFMB, is significantly improved. However, the low CTE characteristics remained insufficient. Additionally, the laminate prepared using this PI film was incompatible with the controlled soft adhesion/easy peeling process. On the other hand, the CpODA/TFMB film (#2R) obtained in this study demonstrated a significantly improved low CTE property, with an improved *σ*_peel_ up to a level close to its compatible range, although there is room for improving film toughness, as shown in Figure 24c. Furthermore, the CpODA(90);BzDAxx(10)/TFMB copolyimide system (#8R, Figure 24d) obviously improved the film toughness, compared with the pristine system, and met nearly all the essential properties at a high level with good balance, including the compatibility with the controlled soft adhesion/easy peel process. Consequently, a promising candidate for innovative plastic substrates for use in flexible displays has been developed.

## 4. Conclusions

In this study, we investigated a semi-cycloaliphatic PI, CpODA/TFMB, which was anticipated to demonstrate superior optical transparency due to inhibited CT interactions and a low CTE property based on its expected main-chain linearity/rigidity. However, the PI film preparation via the conventional two-step process was unsuccessful because of crack formation. Conversely, employing the modified one-pot polymerization at 200 °C using the combined catalyst (1-EP + BA) resulted in a homogeneous and viscous solution of the PI with an extremely high *η*_red_ (4.52 dL/g), yielding a flexible PI cast film. This success hinged on the exceptional solubility of the PI. This study proposes a mechanism to explain the significant catalytic effect on the *M*_w_ enhancement.

Despite the great difficulty in overcoming the trade-off between low CTE and high film toughness, as well as that between low CTE and high solubility, the PI cast film of CpODA/TFMB achieved outstanding overall properties, including very high optical transparency (*T*_400_ = 85.0%, YI = 1.8, Haze = 0.94%), an extremely high *T*_g_ (411 °C), sufficiently high thermal stability (*T*_d_^5^ = 478 °C in N_2_), significantly low CTE (16.7 ppm/K), and acceptable film toughness (*ε*_b_^max^ = 16.3%).

The BzDAxx/TFMB film also exhibited excellent optical transparency, an extremely high *T*_g_, and sufficiently high thermal stability, similarly to the CpODA/TFMB system. The former also showed a moderate CTE (33.1 ppm/K), although not as low as that of CpODA/TFMB. These findings were analyzed based on the steric structures of BzDAxx and CpODA. Interestingly, the BzDAxx/TFMB film showed a remarkably high film toughness (*ε*_b max_ = 49.3%), despite the absence of rotatable ether linkages in the main chains. Taking advantage of this feature, the CpODA/TFMB system was modified by copolymerization with minor contents of BzDAxx (10 and 20 mol%). This approach was effective in improving the film toughness without compromising the low CTE and other desired properties.

The *σ*_peel_ values for the solid–solid laminates comprising the APTES-modified glass substrates and various types of colorless PI films were measured to evaluate the compatibility with the controlled soft adhesion/easy peel process. However, most of the colorless PI films examined were found to be incompatible. Furthermore, no correlation was observed between *σ*_peel_ and the chemical factors related to the PI structures was observed. Therefore, our initial endeavor to identify the PI structural factors (*C*_i_, *C*_F_, and the aromaticity of the imide units) for regulating *σ*_peel_ was unsuccessful. Nonetheless, a good correlation was observed between *σ*_peel_ and CTE of the PI films. This suggests that the adhesion strength in the solid–solid laminates is closely linked to the unexpectedly high chain mobility at the top surface of the PI films. The CpODA(90);BzDAxx(10)/TFMB copolymer demonstrated complete compatibility with the controlled soft adhesion/easy delamination process, while also meeting nearly all other target properties.

Thus, owing to the significant impact of the modified one-pot polymerization method, heat-resistant colorless polymeric materials suitable for plastic substrates in flexible displays were successfully obtained.

## Figures and Tables

**Table 1:** Abbreviations, commercial sources, and melting points of the monomers used in this study.

**Monomer**	**Source**	**Vacuum-Drying Condition**	**Melting Point ^a^ (°C)**
Norbornane-2-spiro-*α*-cyclopentanone-*α*′-spiro-2″-norbornane-5,5″,6,6″-tetracarboxylic dianhydride (CpODA)	ENEOS	160 °C/12 h	333/337 (Double peak)
5,5′-(1,4-Phenylene)-*exo*-bis(hexahydro-4,7- methanoisobenzofuran-*cis-exo*-1,3-dione) (BzDAxx)	ENEOS	160 °C/12 h	209 (broad)
1,2,3,4-Cyclobutanetetracarboxylic dianhydride (CBDA)	Nissan Chemical Industries	180 °C/ 12 h	241
1-*exo*,2-*exo*,4-*exo*,5-*exo*-Cyclohexanetetracarboxylic dianhydride (H-PMDA)	Mitsubishi Gas Chemical New Japan Chemical	160 °C/12 h	303
2,2′-Bis(trifluoromethyl)benzidine (TFMB)	Wakayama Seika	50 °C/12 h	184
4,4′-Oxydianiline (4,4′-ODA)	Wako Chemical	50 °C/24 h	191
2,2-Bis[4-(4-aminophenoxy)phenyl]propane (BAPP)	Wakayama Seika	50 °C/12 h	130

^a^ Data determined from the endothermic peak temperature measured at a heating rate of 5 °C/min on DSC.

**Table 2:** The conditions and results of equimolar polyaddition of CpODA and BzDAxx with aromatic diamines and their film-forming ability.

No.	TCDA	Diamine	Solvent	Solid Content (wt%) Initial → Final	Reaction Period (h)	Appearance of Final Reaction Mixture	*η*_red_ (dL g^−1^)	Appearance of PI Film ^b^
1T	CpODA	4,4′-ODA	NMP	50 → 29.4	72	Homogeneous	0.82 (PAA) 0.76 (PAA) ^a^	non-cracked
2T	ibid	TFMB	DMAc	40 → 30	72	Homogeneous	0.83 (PAA)	cracked
2C	ibid	ibid	ibid	ibid	ibid	ibid	0.71 (PAA) 0.99 (PI)	non-cracked but brittle
3T	BzDAxx	4,4′-ODA	NMP	50 → 31.6	72	Homogeneous	0.62 (PAA)	cracked
4T	ibid	TFMB	DMAc	40 → 30	72	Homogeneous	0.65 (PAA)	cracked
4C	ibid	ibid	ibid	ibid	72	Homogeneous	0.68 (PAA) 0.90 (PI)	cracked

^a^ ref. [50]. ^b^ PI films prepared under the following typical conditions: coating of PAA solutions and drying at 65 °C/3 h in an air convention oven, thermal imidization of the PAA cast films at 200 °C/1 h + 300 °C/1 h under vacuum on a substrate.

**Table 3:** The results of modified one-pot polymerization of CpODA and BzDAxx with aromatic diamines in the presence of the combined catalysts [1-EP (1 Eq.) and BA (1 Eq.)]. The photographs at the bottom of this table show the appearance of the reaction mixtures after modified one-pot process.

No.	TCDA (mol%)	Diamine (mol%)	Solvent	Monomer Content (wt%) Initial → Final	Appearance of Final Reaction Mixture	*η*_red_ (PI) (dL g^−1^)
1R	CpODA	4,4′-ODA	NMP	50.0 → 5.7	Precipitation	---
2R	ibid	TFMB	DMAc	50.0 → 10.8	Homogeneous	4.52
2R′	ibid	ibid	GBL	50.0 → 18.2	Gelation	3.29
5R	ibid	TFMB (90) 4,4′-ODA (10)	NMP	50.0 → 11.0	Homogeneous	3.26
5R′	ibid	ibid	DMAc	50.0 → 8.3	Slightly turbid	3.86
6R	ibid	TFMB (70) BAPP (30)	DMAc	50.0 → 19.7	Homogeneous	2.06
3R	BzDAxx	4,4′-ODA	NMP	50.0 → 4.2	Precipitation	---
4R	ibid	TFMB	GBL	50.0 → 9.9	Homogeneous	4.00
7R	BzDAxx	TFMB (90) 4,4′-ODA (10)	DMAc	50.0 → 9.0	Homogeneous	1.49
8R	CpODA (90) BzDAxx (10)	TFMB	DMAc	50.0 → 18.1	Homogeneous	1.76
9R	CpODA (80) BzDAxx (20)	TFMB	DMAc	50.0 → 15.9	Homogeneous	3.07
10R	CpODA (70) BzDAxx (30)	TFMB	DMAc	50.0 → 15.4	Homogeneous	2.97
11R	CpODA (60) BzDAxx (40)	TFMB	DMAc	30.0 → 9.6	Homogeneous	4.29
12R	CpODA (50) BzDAxx (50)	TFMB	DMAc	50.0 → 8.4	Homogeneous	2.35
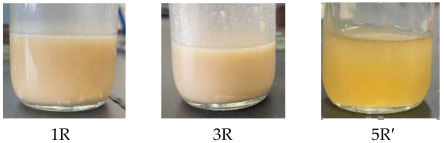						

**Table 4:** Film properties of CpODA- and BzDAxx-based PIs and related systems obtained via the conventional two-step process (T), chemical imidization process (C), and modified one-pot process (R) using combined catalysts (1-EP + BA). The symbols “T”, “C”, and “R” attached to each sample number represent the polymerization and film preparation routes. The photographs at the bottom of this table show the appearance of selected PI films.

No.	TCDA	Diamine (mol%)	*η*_red_ (dL g^−1^)	*T*_400_ (%)	YI	*λ*cut (nm)	*T*_tot_ (%)	Haze (%)	Δ*n*_th_	*ε*_opt_^g^	*T*_g_ (ºC)	CTE (ppm K^−1^)	*E* (GPa)	*ε*_b_ ave/max (%)	*σ*_b_ (GPa)	*T*_d_^5^ (N_2_) (°C)	*T*_d_^5^ (Air) (°C)
1T	CpODA	4,4′-ODA	0.82 ^a^	66.8	9.5	293	86.9	0.92	0.007	2.82	332 ^c^ 360 ^d^	42.0	2.01	11.5/15.9	0.064	485	424
2C	ibid	TFMB	0.71 ^a^ 0.99 ^b^	87.0	1.7	290	90.4	0.71	0.047	2.66	341 ^c^ 336 ^d^	23.7	--- ^e^	--- ^e^	--- ^e^	473	421
2R	ibid	ibid	4.52 ^b^	85.0	1.8	292	90.3	0.94	0.045	2.65	411 ^c^ 410 ^d^	16.7	3.74	9.4/16.3 12.5/23.5 ^f^	0.089	478	432
5R	ibid	TFMB (90) 4,4′-ODA (10)	3.26 ^b^	77.4	6.0	294	88.9	1.29	0.038	2.66	400 ^c^ 419 ^d^	19.2	3.32	8.7/18.8	0.109	483	425
6R	ibid	TFMB (70) BAPP (30)	2.06 ^b^	87.5	1.3	292	90.1	0.64	---	---	362 ^c^ 360 ^d^	26.3	2.56	19.6/34.1	0.105	---	---
4R	BzDAxx	TFMB	4.00 ^b^	86.5	1.4	287	90.3	1.14	---	---	372 ^c^ 374 ^d^	33.1	1.53	13.9/49.3	0.053	472	430
7R	ibid	TFMB (90) 4,4′-ODA (10)	1.49 ^b^	80.4	4.1	289	89.9	1.20	0.029	2.69	368 ^c^ 370 ^d^	36.6	1.98	20.5/59.3	0.078	456	414
8R	CpODA (90) BzDAxx (10)	TFMB	1.76 ^b^	87.0	1.8	295	90.4	1.79	---	---	385 ^d^	20.3	2.24	13.0/28.9	0.081	463	---
9R	CpODA (80) BzDAxx (20)	ibid	3.07 ^b^	86.3	1.8	294	90.4	0.92	---	---	393 ^d^	21.1	2.20	20.0/37.3	0.098	465	---
10R	CpODA (70) BzDAxx (30)	ibid	2.97 ^b^	87.3	1.9	294	90.6	0.82	---	---	411 ^d^	23.7	2.26	11.5/25.0	0.085	469	---
11R	CpODA (60) BzDAxx (40)	ibid	4.29 ^b^	85.8	1.7	291	90.4	1.00	0.038	2.65	390 ^c^ 403 ^d^	25.3	2.13	11.8/24.6	0.076	462	408
12R	CpODA (50) BzDAxx (50)	ibid	2.35 ^b^	86.1	1.9	291	90.4	1.01	0.031	2.64	383 ^c^ 394 ^d^	26.4	2.67	17.0/43.6	0.107	461	414

^a^ Data for PAAs. ^b^ Data for PIs. ^c^ Data measured using DMA (5 °C min^−1^, 0.1 Hz). ^d^ Data measured using TMA (5 °C min^−1^). ^e^ Unavailable data because of the film brittleness. ^f^ Champion data.

**Table 5:** Average peel strengths (*σ*_peel_) and the related data for the laminate samples of PI films and surface-modified glass substrates.

No.	TCDAs	Diamines	*C*_i_ (wt%)	*C*_F_ (wt%)	*E* (GPa)	CTE (ppm/K)	*σ*_peel_ (N/cm)	Process Compatibility Rank (1–5)
2R	CpODA	TFMB	20.95	17.05	3.74	16.7	0.10	3.5
8R	CpODA (90) BzDAxx (10)	TFMB	20.88	16.99	2.24	20.3	0.21	5
9R	CpODA (80) BzDAxx (20)	TFMB	20.81	16.94	2.20	21.1	0.51	3
10R	CpODA (70) BzDAxx (30)	TFMB	20.74	16.88	2.26	23.7	0.64	3
4R	BzDAxx	TFMB	20.28	16.50	1.53	33.1	1.11	1
13R	H-PMDA	TFMB	27.55	22.42	3.13 ^a^	57.1 ^a^	3.58	1
14T	ODPA	TFMB	23.56	19.18	3.39 ^b^	63.8 ^b^	3.41	1
15C	6FDA	TFMB	19.23	31.30	3.05 ^c^	52.9 ^c^	1.68	1
16T	*s*-BPDA	TFMB	24.21	19.71	3.67 ^d^	33.8 ^d^	0.14	4
17T	*s*-BPDA	M-APAB	27.99	0	9.38 ^e^	6.5 ^e^	0.13	4
18	XENOMAX_®_ film		---	---	9.5 ^f^	3.0 ^g^	0.20	5

^a^ Ref. [56], ^b^ Data measured in our group, ^c^ Ref. [30], ^d^ Ref. [96], ^e^ Ref. [100], ^f^ Ref. [97], ^g^ Ref. [66].

**Table 6:** Criteria established for ranking the achievement level for each target property.

Properties	Parameters	Ranking				
		1	2	3	4	5
Physical heat resistance (HR)	*T*_g_ (°C)	≤ 200	220–240	250–270	280–300	≥360 or unclear *T*_g_ by DMA
Low CTE property (LCTE)	CTE (ppm K^−1^)	≥70	60–50	45–35	30–20	≤10
Light transmittance (Tr)	*T*_400_ (%)	≤5	20–30	40–60	70–80	≥85
Low yellowness (LY)	YI	≥20	10–7	6–4	3–2	≤1
Low haze (LH)	Haze (%)	≥10	4–3	3–2	2–1	≤1
Toughness (To)	*ε*_b max_ (%)	≤2 or no film-forming ability	5–10	20–30	40–60	≥80
Solution processability (SP)	Qualitative solubility	Insoluble	Soluble in amide solvents (≤1 wt%)	Soluble in amide solvents (3–5 wt%)	Soluble in amide solvents (>~10 wt%)	Soluble in non-amide solvents (>~10wt%)
Controlled soft adhesion/easy removability (S/E)	Peel strength (N/cm)	<0.01 or >1.0	---	0.05–0.08 or 0.40–0.60	---	0.15–0.30

## Data Availability

The data supporting this study are available within the article and/or in the Supplementary Materials.

## Supplementary Materials

The following supporting information can be downloaded at: https://www.mdpi.com/article/10.3390/polym17131887/s1, Document S1: The synthetic procedures and analytical results of CpODA-based diimide model compound. Document S2: The synthetic procedures and analytical results of BzDAxx-based diimide model compound. Scheme S1: Molecular structure of CpODA-based model compound with numbering. Scheme S2: Molecular structure of BzDAxx-based model compound with numbering. Figure S1: FT-IR transmission spectrum of thin cast film for CpODA/TFMB obtained via modified one-pot polymerization. Figure S2: DMA curves for the CpODA/TFMB polyimide film (#2R). Figure S3: TMA curve for the CpODA/TFMB polyimide film (#2R). Figure S4: Peel strength curve during the peel testing for a laminate sample between XENOMAX_®_ film and glass substrate. Figure S5: Reaction route for the synthesis of *trans*- and *cis-exo-exo* CpODA mixture [78]. Figure S6: Reaction route for the synthesis of BzDAxx [79]. Figure S7: Correlation of peel strength and parameters relating to the chemical composition in the PIs: (a) imide group content (*C*_i_) and (b) fluorine group content (*C*_F_).
