# Supplementary material for: Colorless Polyimides with Low Linear Coefficients of Thermal Expansion and Their Controlled Soft Adhesion/Easy Removability on Glass Substrates: Role of Modified One-Pot Polymerization Method
Source: Polymers (Basel). 2025 Jul 7;17(13):1887. doi: 10.3390/polym17131887 (PMC12252339; doi:10.3390/polym17131887)
Supplement: Supplementary file 1 [file polymers-17-01887-s001.zip › polymers-3725992-supplementary.pdf]

Supplementary Materials (Polymers-3725992, R1)

Document S1. The synthetic procedures and analytical results of CpODA-based diimide model compound.

In a three-necked 100 mL-flask, CpODA (3.0 mmol) and aniline (8.0 mmol) were dissolved in dehydrated *N*,*N*-dimethylacetamide (DMAc, 8.1 mL), and the reaction mixture was refluxed at 160 ºC for 4 h in an N_2 atmosphere while gradually adding DMAc (additionally, 10.1 mL). After cooling to room temperature, the white precipitate formed was collected by filtration, washed with methanol, and dried at 100 ºC for 12 h under vacuum (yield: 75%). The molecular structure of this product (**Scheme S1**) was confirmed by the following data. FT-IR (KBr plate method, cm^{-1}): 3066 (C_{arom}–H stretching), 2984/2959/2883 (C_{aliph}–H), 1771/1719 (imide, C=O). 1493 (phenyl), 1375 (imide, N–Ph), 747 (imide, ring deformation). ^{1}H-NMR (400 MHz, DMSO-d_6, δ, ppm): 7.49 [t, 4H (4.00H), J = 7.3 Hz, 3,3′,5,5′-protons of the terminal aniline (AN) unit], 7.43 [d, 2H (2.09H), J = 7.4 Hz, 4,4′-protons of AN], 7.25 [d, 4H (3.99H), J = 7.1 Hz, 2,2′,6,6′-protons of AN], 3.11 [d, 2H (2.15H), J = 7.1 Hz, 3,3′-protons of NB], 2.98 [d, 2H (2.13H), J = 7.2 Hz, 2,2′-protons of NB], 2.63 [d, 2H (2.04H), J = 3.4 Hz, 4,4′-protons of NB], 2.47 [s, 2H (2.16H), 1,1′-protons of NB], 2.09 [d, 2H (2.13H), J = 10.9 Hz, 6(H_a),6′(H_a)-protons of NB], 1.89 [s, 4H, the cyclopentane unit], 1.84 [dd, 2H, J = 12.3, 4.3 Hz, 7(H_a),7′(H_a)-protons of NB], 1.44 [d, 2H (2.11H), J = 12.4 Hz, 7(H_b),7′(H_b)-protons of NB], 1.31 [d, 2H (2.00H), J = 10.9 Hz, 6(H_b),6′(H_b)-protons of NB].

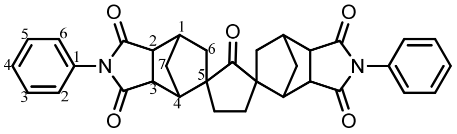

Scheme S1. Molecular structure of CpODA-based model compound with numbering.

Document S2. The synthetic procedures and analytical results of BzDAxx-based diimide model compound.

BzDAxx-based diimide model compound was synthesized from BzDAxx and aniline in a similar manner (yield: 33%). The molecular structure of this product (**Scheme 4**) was confirmed by the following data. FT-IR (KBr plate method, cm^{-1}): 3057 (C_{arom}–H stretching), 2965/2886 (C_{aliph}–H), 1775/1710(imide, C=O). 1498 (phenyl), 1389 (imide, N–Ph), 752 (imide, ring deformation). ^{1}H-NMR (400 MHz, DMSO-d_6, δ, ppm): 7.49 [t, 4H (4.00H), J = 7.4 Hz, 3,3′,5,5′-protons of the terminal aniline (AN) unit], 7.43 [d, 2H (1.99H), J = 7.5 Hz, 4,4′-protons of AN], 7.26 [d, 4H (4.18H), J = 8.2 Hz, 2,2′,6,6′-protons of AN], 7.24 [s, 4H (4.03H), the central 1,4-phenylen unit], 3.09 [d, 2H (2.18H), J = 7.2 Hz, 3,3′-protons of the norbornane (NB) units], 3.00–2.95 [m, 4H (4.07H), 2,2′- + 5,5′-protons of NB], 2.97–2.93 [m, 2H (2.02H), 5,5′-protons of NB], 2.72 [d, 2H (2.03H), 4,4′-protons of NB], 2.62 [s, 2H (2.08H), 1,1′-protons of NB], 1.96–1.94 [m, 2H (2.06H), 6(H_a),6′(H_a)-protons of NB], 1.83–1.80 [m, 2H (1.98H), 6(H_b),6′(H_b)-protons of NB], 1.52 [d, 2H (2.01H), J = 11.3 Hz, 7(H_a),7′(H_a)-protons of NB], 1.26 [d, 2H (2.06H), J = 11.1 Hz, 7(H_b),7′(H_b)-protons of NB].

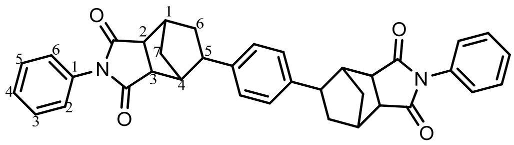

Scheme S2. Molecular structure of BzDAxx-based model compound with numbering.

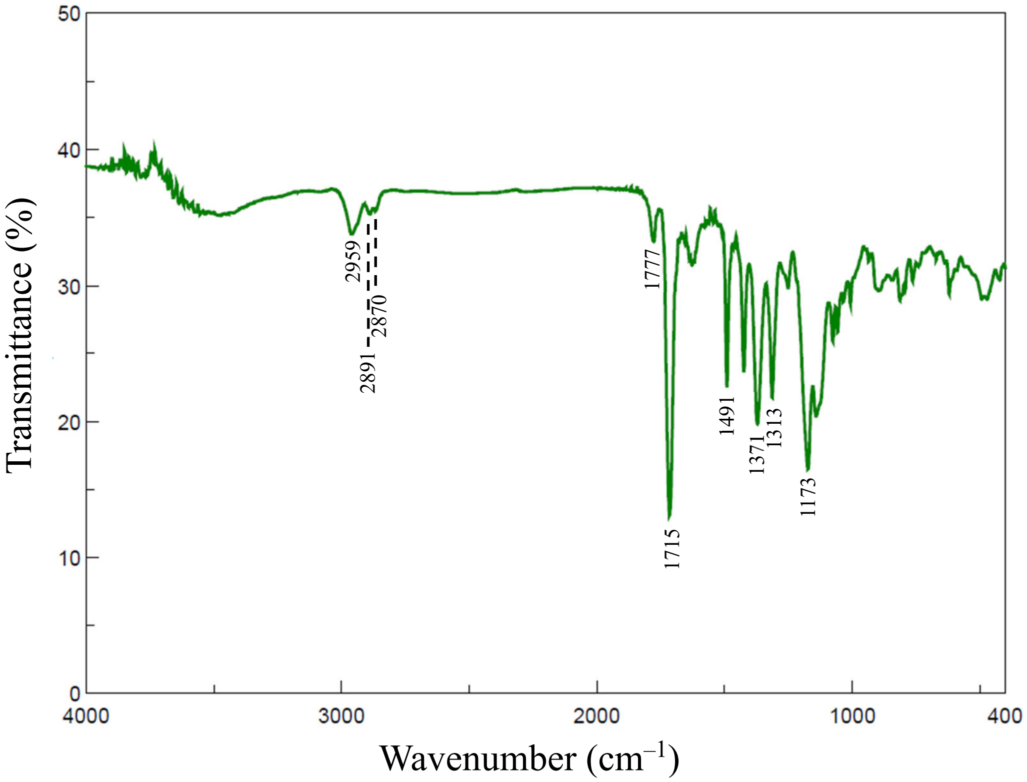

Figure S1. FT-IR transmission spectrum of thin cast film for CpODA/TFMB obtained via modified one-pot polymerization.

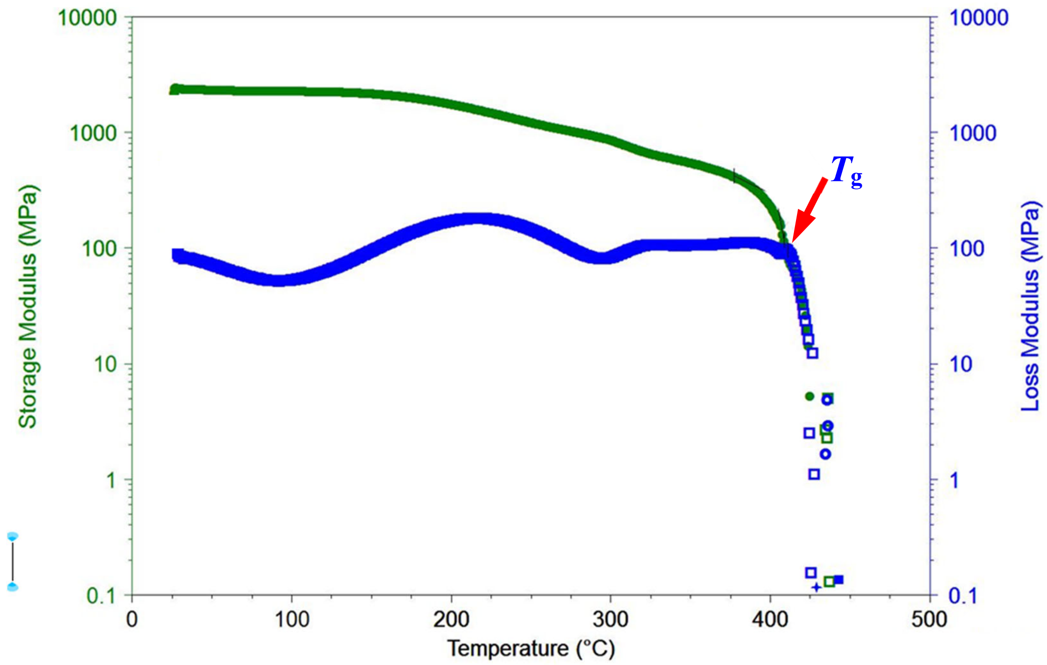

Figure S2. DMA curves for the CpODA/TFMB polyimide film (**#2R**).

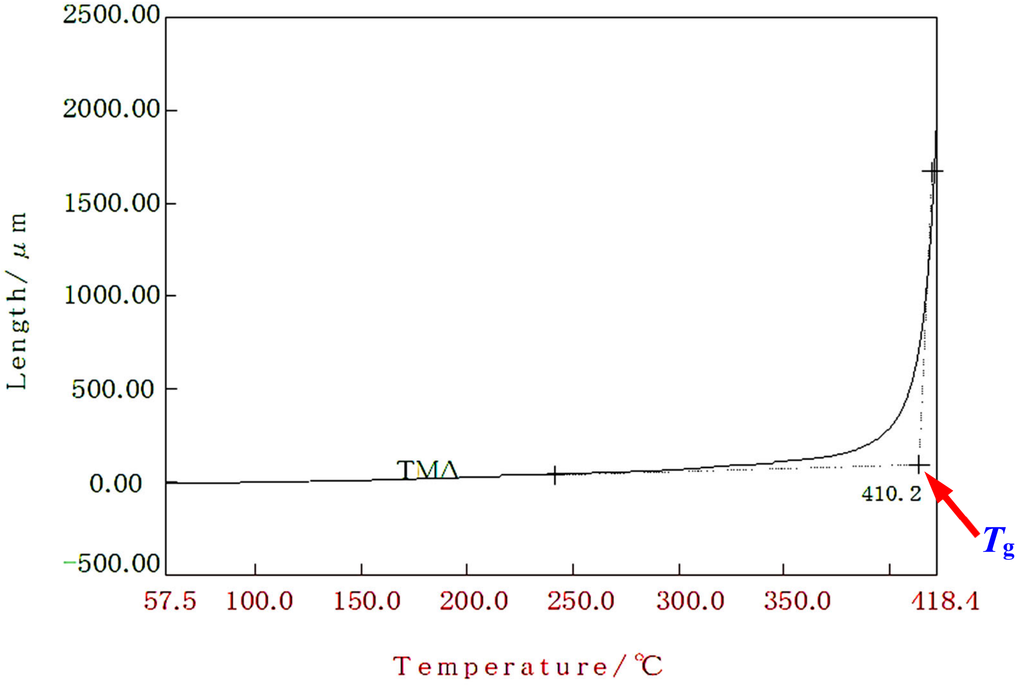

Figure S3. TMA curve for the CpODA/TFMB polyimide film (**#2R**).

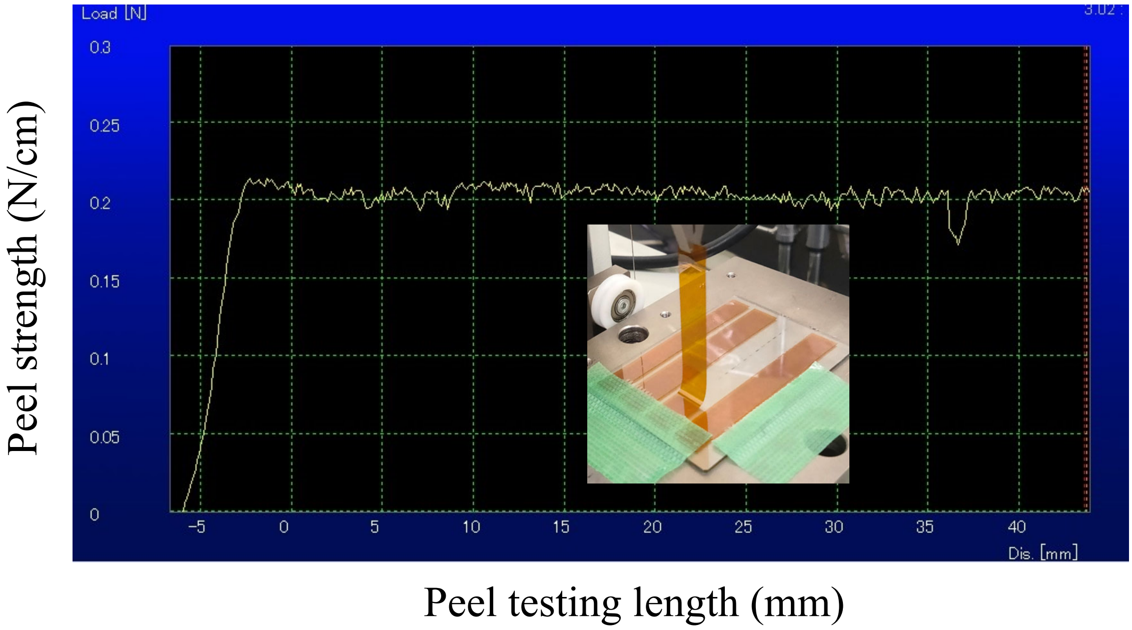

Figure S4. Peel strength curve during the peel testing for a laminate sample between XENOMAX® film and glass substrate.

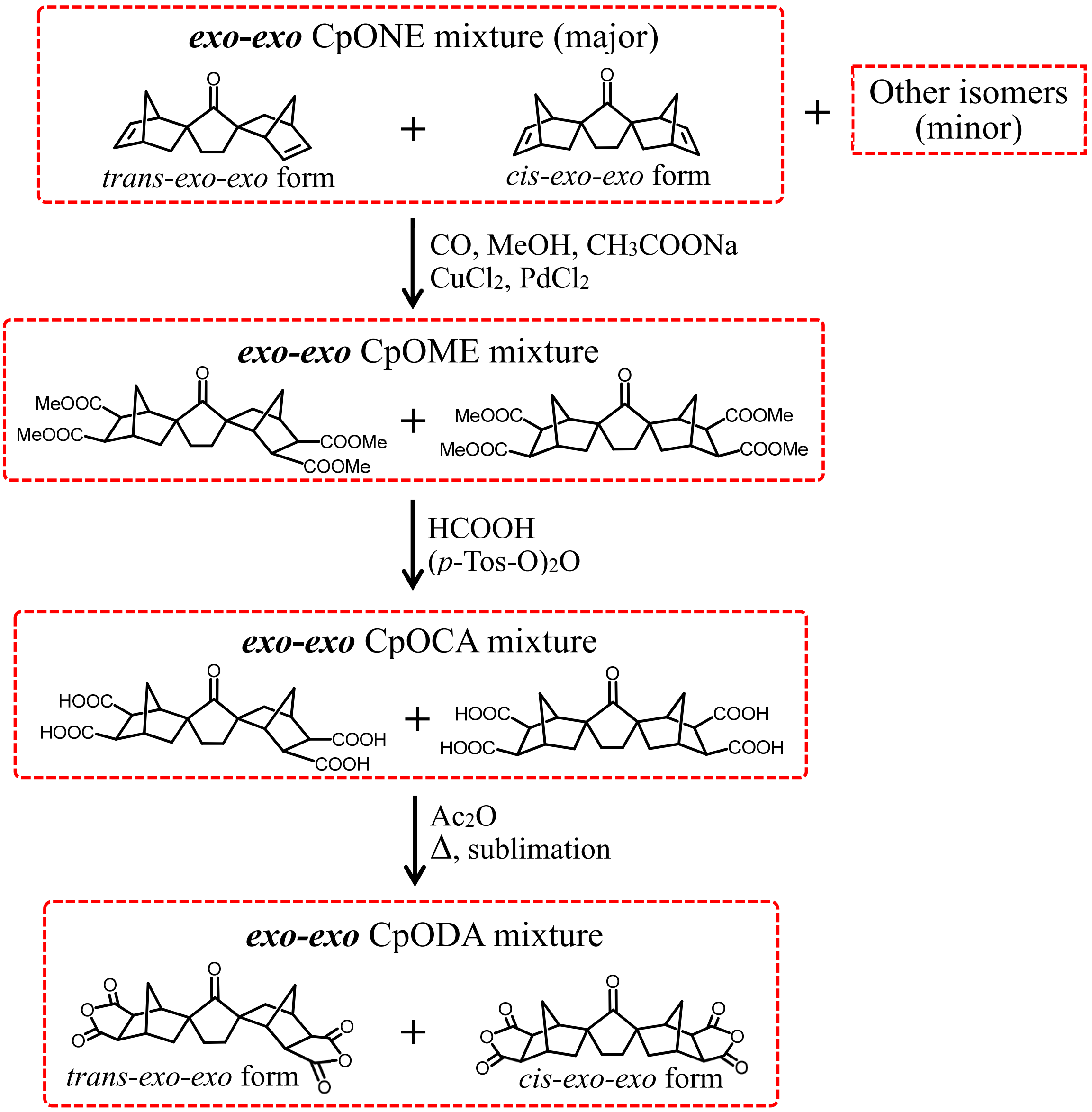

Figure S5. Reaction route for the synthesis of *trans*- and *cis*-*exo*-*exo* CpODA mixture [**78**].

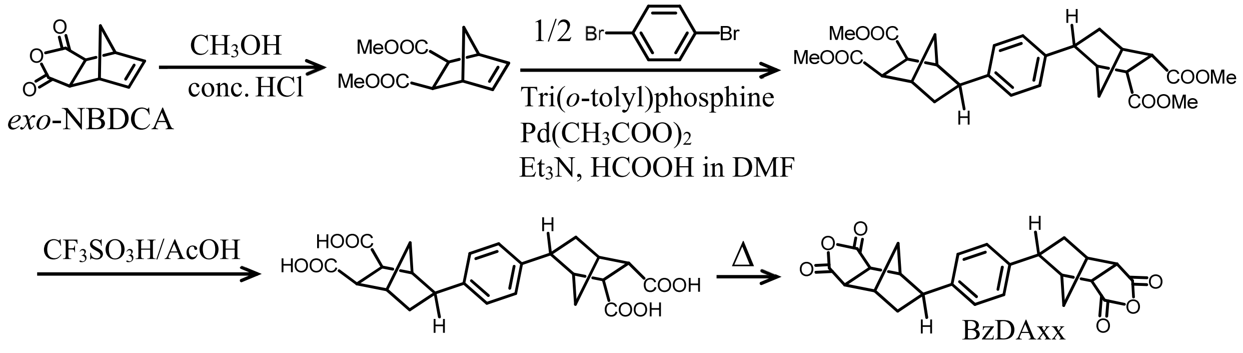

Figure S6. Reaction route for the synthesis of BzDAxx [**79**].

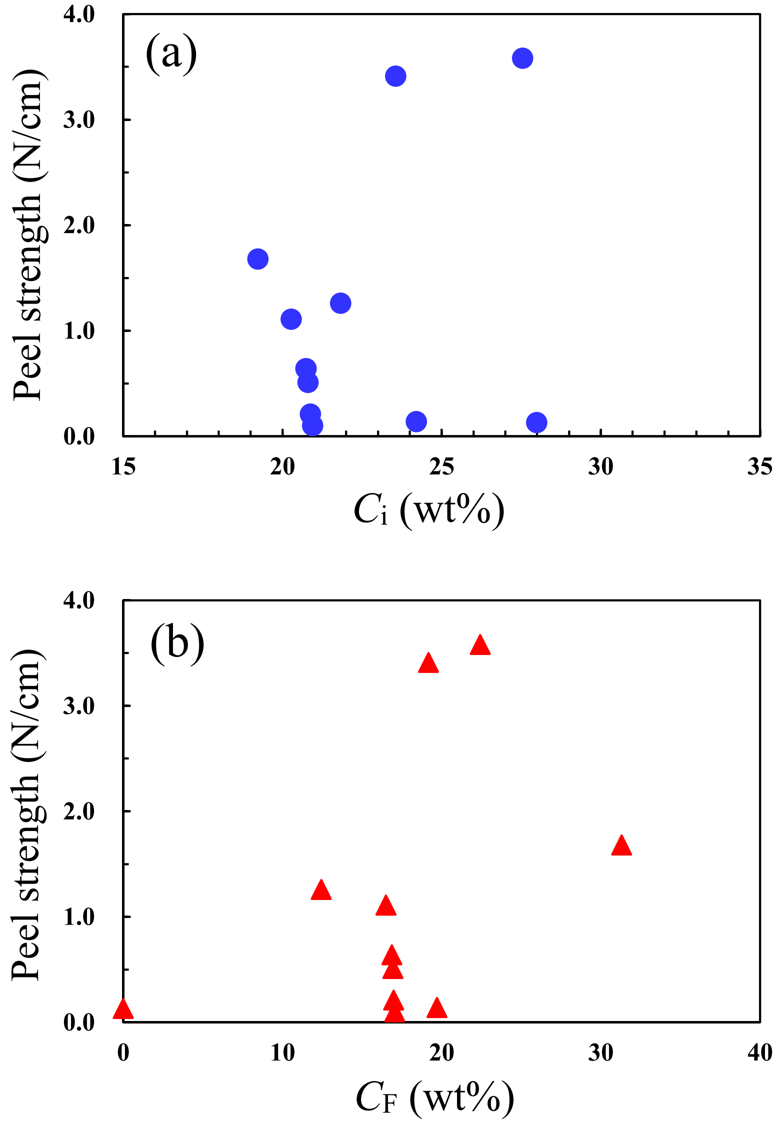

Figure S7. Correlation of peel strength and parameters relating to the chemical composition in the PIs: (a) imide group content and (b) fluorine group content.
